# An Overview of Biofilm Formation–Combating Strategies and Mechanisms of Action of Antibiofilm Agents

**DOI:** 10.3390/life12081110

**Published:** 2022-07-23

**Authors:** Syeda Tasmia Asma, Kálmán Imre, Adriana Morar, Viorel Herman, Ulas Acaroz, Hamid Mukhtar, Damla Arslan-Acaroz, Syed Rizwan Ali Shah, Robin Gerlach

**Affiliations:** 1 Department of Food Hygiene and Technology, Faculty of Veterinary Medicine, Afyon Kocatepe University, Afyonkarahisar 03200, Turkey; tasmiaasma@gmail.com (S.T.A.); ulasacaroz@hotmail.com (U.A.); 2 Department of Animal Production and Veterinary Public Health, Faculty of Veterinary Medicine, Banat’s University of Agricultural Sciences and Veterinary Medicine “King Michael I of Romania”, 300645 Timisoara, Romania; adrianamo2001@yahoo.com; 3 Department of Infectious Diseases and Preventive Medicine, Faculty of Veterinary Medicine, Banat’s University of Agricultural Sciences and Veterinary Medicine “King Michael I of Romania”, 300645 Timisoara, Romania; viorel.herman@fmvt.ro; 4 Institute of Industrial Biotechnology (IIB), Government College University (GCU), Lahore 54000, Pakistan; hamidmukhtar@gcu.edu.pk; 5 Department of Biochemistry, Faculty of Veterinary Medicine, Afyon Kocatepe University, Afyonkarahisar 03200, Turkey; damlaarslan06@hotmail.com; 6 Department of Animal Nutrition and Nutritional Diseases, Faculty of Veterinary Medicine, Afyon Kocatepe University, Afyonkarahisar 03200, Turkey; rizwansyed604@gmail.com; 7 Department of Biological and Chemical Engineering, Montana State University, Bozeman, MT 59717, USA; robin_g@montana.edu

**Keywords:** biofilms, antibiotic resistance, combating strategies, antibiofilm agents, natural products, antibodies, nanomaterials

## Abstract

Biofilm formation on surfaces via microbial colonization causes infections and has become a major health issue globally. The biofilm lifestyle provides resistance to environmental stresses and antimicrobial therapies. Biofilms can cause several chronic conditions, and effective treatment has become a challenge due to increased antimicrobial resistance. Antibiotics available for treating biofilm-associated infections are generally not very effective and require high doses that may cause toxicity in the host. Therefore, it is essential to study and develop efficient anti-biofilm strategies that can significantly reduce the rate of biofilm-associated healthcare problems. In this context, some effective combating strategies with potential anti-biofilm agents, including plant extracts, peptides, enzymes, lantibiotics, chelating agents, biosurfactants, polysaccharides, organic, inorganic, and metal nanoparticles, etc., have been reviewed to overcome biofilm-associated healthcare problems. From their extensive literature survey, it can be concluded that these molecules with considerable structural alterations might be applied to the treatment of biofilm-associated infections, by evaluating their significant delivery to the target site of the host. To design effective anti-biofilm molecules, it must be assured that the minimum inhibitory concentrations of these anti-biofilm compounds can eradicate biofilm-associated infections without causing toxic effects at a significant rate.

## 1. Introduction

Biofilms are referred to as the complex and sessile communities of microorganisms in aggregate forms either adhered to any surface or concealed in an extracellular matrix [1]. Complex aggregates of microbes in the form of biofilms enable them to tolerate harsh environmental conditions such as desiccation and starvation [2]. Consequently, it is believed to be the emerging root of different nosocomial infections in patients with immunodeficiency [3]. Medical treatment devices such as contact lenses, catheters, prosthetic heart-valves, cardiac pacemakers, dentures, and joint prosthesis may provide the desired surfaces for biofilm formation. Approximately 50% of the nosocomial infections reported in immunodeficient patients are caused by biofilms [4,5]. In the case of implants, a considerable rise in biofilm development has been observed [6]. In some cases, antibiotics such as colistin, imipenem, and many others can mitigate. However, they cannot eradicate the whole biofilm with low concentration, and an increase in concentration can cause severe side effects and toxicity [7,8]. The higher concentrations of minimum bactericidal concentration (MBC) and minimum inhibitory concentration (MIC) of antibiotics required for the microbial cells of biofilms make the treatment less effective [9].

Furthermore, biofilms shield the invading bacterial cells against the host’s immune system by the weakened phagocytic activity and the complement system [10,11] or by making them more resistant to conventionally used antibiotics [12,13,14]. Several factors are known to be responsible for resistance development, such as structure and nature of biofilm, oxygen and nutrient accessibility, metabolic state, inherent and acquired microbial resistance, etc. Literature has shown that the mucoid nature of *Pseudomonas aeruginosa* biofilm made it highly resistant to tobramycin [15]. Cells growing slowly with depleted nutrients such as the stationary phase cells are also insensitive to certain antibiotics, while actively growing or dividing cells can be sensitive to antibiotics such as *β*-lactams [16,17].

Walters et al. showed that limited oxygen can influence the antibiotic resistance in *P. aeruginosa,* and reported that the antibiotic was effective only in the oxygenated portion of the biofilm (within 50–90 µm) of the air–biofilm interface [18]. Furthermore, studies indicated that when the bacterial cells building the biofilms are exposed to high concentrations of antibiotics, they can experience a higher mutation rate than planktonic bacteria with a 10-fold escalation in plasmid-mediated resistance [19]. Some mucoid biofilms do not directly adhere to the surface; instead, they form aggregates by attaching or remaining attached to neighboring microbes inside a biologically produced polymeric matrix. This has been observed in cystic fibrosis patients, where mucoid biofilms were found not attached to the lung tissue [20]. These and other findings demonstrated that surface-adhered and non-surface-attached microbial communities or aggregates can have similar tolerances against polymorphonuclear leukocytes and certain antibiotics. These bacterial aggregates have been known to cause wound, middle ear, and different chronic infections [21,22,23]. Recently, Wu et al. published a study on biofilm combating strategies such as by replacing the infected implants and by removing the infected foreign bodies (e.g., stents), cyclic di-GMP modification, and quorum sensing (QS) inhibition [4]. There is an ongoing concerted effort to develop new anti-biofilm compounds and effective drugs for combating biofilms and their associated infections. This review mainly focuses on established biofilm inhibition targets and effective combating strategies.

## 2. Biofilm Formation

**Figure 1:**
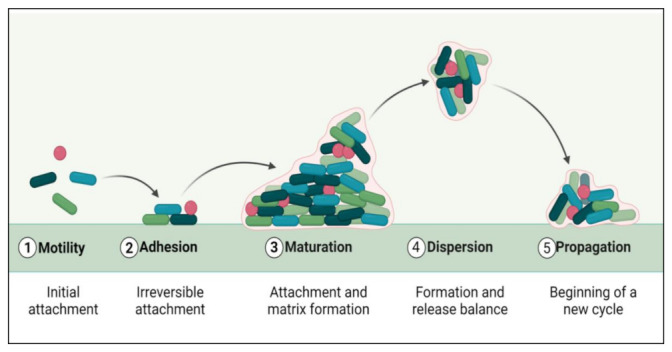
Phases of biofilm formation.

The biofilm formation and development process include five distinct phases (Figure 1). The initial step involves the microbial cells surface adhesion, followed by the growth and formation of mature biofilms [24]. Many factors, such as sedimentation, Van der Waals forces, hydrodynamic forces, Brownian movements, and electrostatic or hydrophobic interactions, mediate bacterial deposition [25]. Biofilm formation involves some surface-linked proteins such as protein A [26], SasG [27,28], fibronectin-binding protein [29], biofilm-associated protein (BAP) [30,31], and OmpA are involved in this initial phase of biofilm development. Some microbial species cannot adhere directly to a surface, but they can bind to cells or a matrix already present. Microbial cells in biofilms are ultimately encased in an extracellular matrix, a variable mixture of biomolecules such as nucleic acids, proteins, lipids, and polysaccharides [32]. Biofilm formation and maturation can be affected by QS, a cell–cell communication mechanism promoted via small signaling molecules [33].

The extracellular biofilm matrix protects the bacterial cells against external stress conditions but does not necessarily create a physical barrier for antimicrobials. The dispersion of biofilm cells can be induced chemically or through mechanical stresses. Anderl et al. demonstrated that ampicillin was able to infiltrate the *β*-lactamase deficient biofilm of *Klebsiella pneumoniae* strain while ampicillin was unable to penetrate the *β*-lactamase owing wild type strain of *K. pneumoniae*, but in the latter case, it was demonstrated that ampicillin was degraded before penetration could occur into the biofilm [34].

## 3. Biofilm Combating Strategies

The lack of effectiveness of conventional therapeutics indicates that biofilm treatment requires additional improvements [35]. Hence, novel strategies are needed to combat biofilm-associated challenges such as resistance to different antibiotics and strong pathogenicity. Foreign bodies, such as implants, are a major factor in developing biofilm-associated infections [36]. Removal or replacement of infected implants or contaminated medical equipment and effective antibiotics seem crucial for treating biofilm-linked infections. The use of long-term antibiotic administration is recommended to prevent biofilm growth if removal or replacement is not possible [37]. Previous studies indicated that the treatment of mature biofilms is often not effective as compared to the treatment of premature biofilms. However, inefficient diagnostic approaches for premature biofilms have allowed the development of certain clinical conditions by forming mature biofilms inside the body [38].

Antibiotics for treating biofilms should be chosen based on their penetration ability and sensitivity toward the biofilm matrix [4]. Previous studies have indicated that planktonic cells are not much resistant to antibiotics than biofilm bacteria [39]. A combination of different antimicrobials with different modes of action is beneficial, for instance, one agent being efficacious against growing microbial cells and other agents against dormant cells [40]. Hence, it suggests applying combinatorial therapy with significant efficacy instead of mono-therapeutic antibiotic treatment. Furthermore, combinatorial therapy also needs an adequate dispensation of antibiotics with proper dosage and time.

More recently, the development of strategies to prevent biofilm development has emerged as an area of interest [41,42]. Polyethylene glycol (PEG), a polymeric hydrophilic coating, is an example of an antimicrobial or antifouling surface that decreases microbial attachment [41,43]. Disinfectant- or antibiotic-impregnation is often required to develop antimicrobial or antifouling surfaces such as polyurethane polymers [43,44]. Nanoparticle (NP) coatings such as silver NPs with antioxidant and antibacterial properties can also be applied to prevent biofilm developments [45,46]. However, surface coatings face challenges due to erosion or leaching, which might allow for successful cell attachment, survival, and biofilm development.

Developing significant anti-biofilm compounds or biofilm dispersal methods is another emerging strategy to control or destroy potentially harmful biofilms [47]. Several molecules can be used as anti-biofilm agents, these include peptides, enzymes, polyphenols, certain antibiotics, etc. [48]. Some of these anti-biofilm agents impede Gram-positive and negative bacterial signaling pathways; some anti-biofilm agents, along with susceptible microbes, are listed in Table 1.

## 4. Mechanism of Action of Different Anti-Biofilm Agents

Anti-biofilm agents belonging to various active molecules can cause biofilm inhibition and degradation. Several agents have been known to have anti-biofilm activity, including some natural products, synthetic compounds, enzymes, peptides, chelating agents, polyphenols, and some antibiotics. These anti-biofilm agents have different modes of action against various bacteria to inhibit biofilm development, as tabulated in Table 2.

### 4.1. AHL-Mediated QS Inhibition

Quorum sensing (QS) is a system of communication between bacterial cells, through the activation of specific signals, with the main purpose of facilitating the adaptation of bacteria to the adverse environmental conditions, including bacterial population densities. This process involves synthesizing, sensing and reacting to extracellular chemical signaling molecules, so-called autoinducers (AIs). Gram-negative bacteria communicate using AIs, most commonly acyl-homoserine lactones (AHLs) or other small molecules.

**Figure 2:**
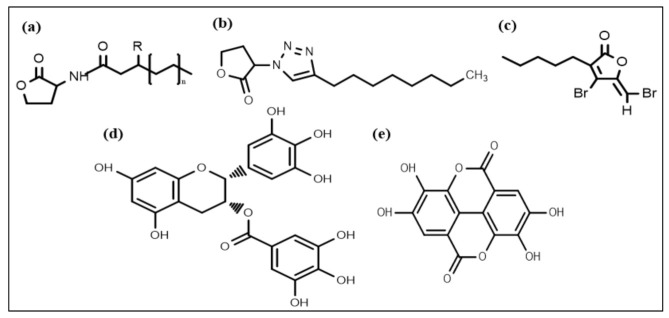
Chemical structures of some anti-biofilm compounds that inhibit AHL-mediated QS. (**a**) AHL, (**b**) triazole dihydro furanone, (**c**) synthetic halogenated furanone, (**d**) EGCG, (**e**) ellagic acid.

Nowadays, considerable effort is focused on developing new chemical strategies to disrupt these signals and mitigate quorum sensing controlled responses for biofilm control. In this regard, the most promising developed anti-biofilm chemical structures include N-acyl homoserine lactones (AHL) (Figure 2a), triazole dihydro furanone (Figure 2b), synthetic halogenated furanone (Figure 2c), EGCG (Figure 2d), and ellagic acid (Figure 2e), respectively. Many N-acyl homoserine lactones (AHL) analogs have been prepared by altering the lactone ring or the acyl side chain [98,99,100,101,102,103], and a few AHL analogs have been developed by modifying amide moieties [104,105,106]. Several AHLs were observed to disrupt biofilm formation. Cyclohexanone or cyclopentyl replacement to the lactone moiety of the native AHL molecules has shown a significant biofilm inhibitory effect against *P. aeruginosa* and *S. marcescens* [107,108]. AHL analog, in which the amide moiety was altered by triazole dihydro furanone, exhibited biofilm inhibitory as well as biofilm eliminating activity against *P. aeruginosa* and *Burkholderia cenocepacia* [106]. AHL analogs with phenoxyacetyl and phenylpropionyl homoserine lactones (aromatic groups) replacements on the acyl side chain showed inhibition of *P. aeruginosa* biofilm development [100,109].

**Figure 3:**
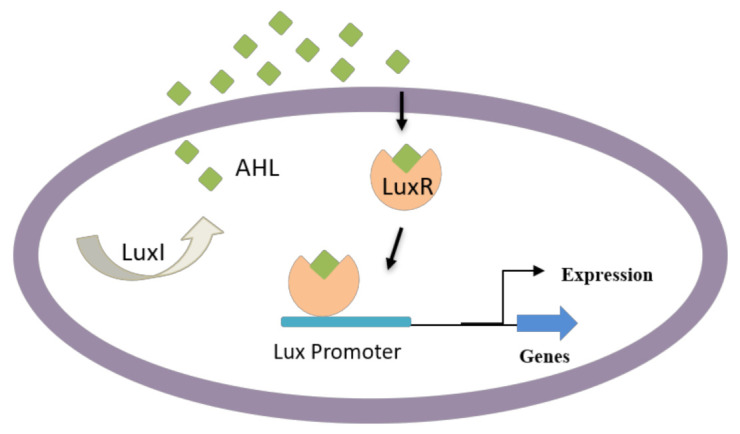
QS in Gram-negative bacteria; some bacteria can secrete AHL that enters neighboring cells and induce the QS-mediated formation of virulence factors and biofilm development.

Apart from AHL-resembling compounds, numerous other compounds have shown blocking of AHL-mediated QS, resulting in biofilm disruption. Some of these compounds are produced naturally, including dihydroxybergamottin and bergamottin from plant extracts (e.g., grapefruit juice), which have affected *P. aeruginosa* biofilm formation by inhibiting AHL-mediated QS [110,111]. AHL-dependent QS is known to be responsible for regulating several virulence factors in Gram-negative bacteria such as bacterial attachment, biofilm development, pigment production, and exoenzyme secretion [112,113]. Particularly, Gram-negative bacteria use AHL as signaling molecules (Figure 3) in the QS process to manage population density and swarming motility of bacteria. These molecules vary based on acyl side chain substitutions and length and are produced by an enzyme *LuxI*-type synthase [63]. Above specific concentrations, targeted gene expression is regulated by the binding of signaling molecules to cognate *LuxR*-type (transcriptional activator protein) [114,115].

Natural furanone is isolated from an Australian macroalga called *Delisea pulchra* and is used to produce a synthetically halogenated furanone compound [116]. Halogenated furanones can impede bacterial swarming and signaling processes by interrupting the interaction between AHL molecules and putative-regulatory proteins through competitive receptor binding [116]. The furanones has inhibitory properties on aggregation characteristics of ecologically relevant bacterial strains with relevant concentrations surface.

The literature describes that the furanones particularly target the *rhl* system, which incorporates in QS and penetrates the *P. aeruginosa* biofilm matrix, thus disturbing the gene expression associated with QS mediated biofilm maturity. Furanones work by altering the biofilm structure facilitating the detachment of bacteria resulting in the loss of bacterial biomass from the substratum [63]. Furanones have been demonstrated experimentally to have several functions, such as suppression of AHL-dependent bioluminescence expression, inhibition of AHL-directed generation of virulence factors, and QS led to luminescence inhibition [63,117,118,119]. Certain polyphenols (such as ellagic acid, EGCG, and tannic acid) have similar biofilm inhibition mechanisms. However, they require higher concentrations than furanones to achieve similar effects [120].

**Figure 4:**
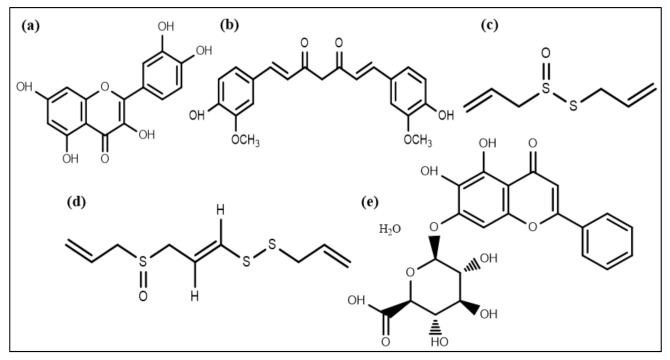
Chemical structures of some anti-biofilm compounds that inhibit AHL-mediated QS. (**a**) quercetin, (**b**) curcumin, (**c**) allicin, (**d**) ajoene, (**e**) baicalin hydrate.

A flavonoid compound, quercetin (Figure 4a), also affects QS and acts as an antibiofilm molecule against *S. aureus* via inhibiting alginate production resulting in decreased attachment in biofilm formation. It is also responsible for reducing exopolysaccharide production (EPS), which is required for bacterial attachment and biofilm formation [121]. In addition to quercetin, two other synthetic flavonoids are also recognized as anti-biofilm agents by dispersing cells of *S. aureus* [122]. A phytochemical, curcumin (Figure 4b), isolated from *Curcuma longa,* manifests potential anti-biofilm activity by influencing the gene expression involved in the QS process and thus the development of virulence factors such as swarming movement and the production of alginate [123].

Extracts of *Allium sativum* (garlic) and different *Penicillium* species have some components that can inhibit QS [124]. Patulin and penicillanic acid extracted from *Penicillium* species responsible for QS inhibition were found using mass spectrometry and chromatography techniques [125]. Allicin and ajoene (a cyclic thioacetal and cyclic disulfide) (Figure 4c,d) extracted from garlic were also found to be QS inhibiting compounds [126]. Garlic extracts and patulin escalated *P. aeruginosa* biofilm susceptibility towards the antibiotic tobramycin, resulting in magnified *P. aeruginosa* biofilms clearance in an in vivo pulmonary infected model [124,127,128]. Moreover, various phenolic compounds, including epigallocatechin and baicalin hydrate (Figure 4e), occluded AHL-mediated QS [120,129]. Furthermore, these compounds exhibited no effect on microbial adhesion but disrupted biofilm development of *P. aeruginosa*, *Burkholderia multivorans,* and *B. cenocepacia* at upcoming stages of biofilm expansion and its maturation [120,130].

### 4.2. Membrane Permeabilization or Potential Alteration

**Figure 5:**
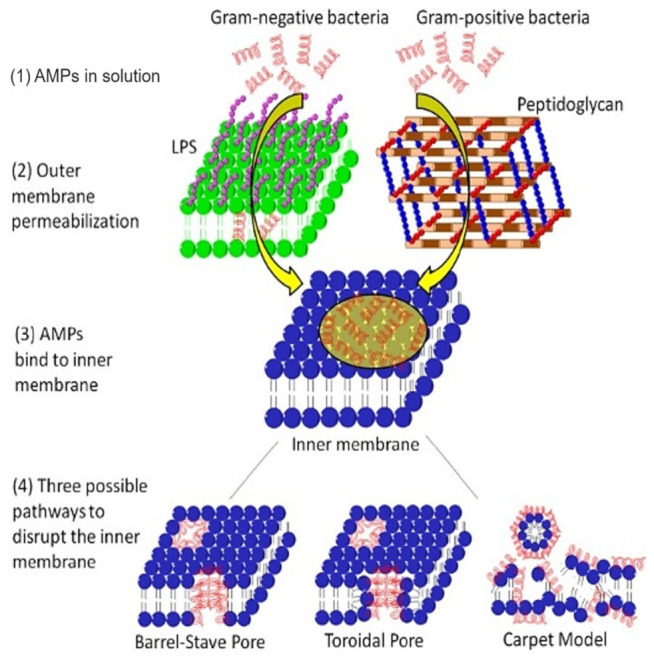
Mechanism of action of AMPs on the membrane system of Gram-negative and Gram-positive bacteria. In Gram-negative bacteria, the AMP outreach the cytoplasmic membrane via permeabilizing the outer membrane, while in Gram-positive bacteria, the AMP directly disperses through nano ranged pores of the peptidoglycan layer. After binding to the inner membrane, APMs can create three types of pores (barrel-stave pore, toroidal pore, or carpet model). Adapted from Jianguo et al. 2017, [131].

The alteration of bacterial membrane permeability results in pore formation and destruction of the cytoplasmic membrane. Antimicrobial peptides (AMPs) disrupt bacterial membranes via three possible mechanisms: (i) pore-induced barrel-stave pathway, (ii) toroidal pathway, or (iii) carpet (non-pore) mode (Figure 5) [131]. The lantibiotics are ring-structured peptide antibiotics containing thioether amino acids (methyllanthionine or lanthionine) or unsaturated amino acids (2-amino isobutyric acids or dehydro-alanine). These peptides are produced and post-translationally modified, and inhibit bacteria by disrupting their membranes, consequently inhibiting enzyme production [132]. Subtilin, a significant (pore-forming) lantibiotic produced from a Gram-positive bacteria *B. subtilis* (ATCC 6633 strain), induces the dissipation of transmembrane electrostatic-potential releasing cytoplasmic solutes from *B. subtilis* and *Staphylococcus simulans* membrane vesicles [49]. Nisin, the most popular lantibiotic and structurally similar to subtilin, inhibits the biosynthesis of the cell wall by complexing with lipid-I and lipid-II [133,134]. Nisin can permeabilize the cytoplasmic membrane via ephemeral pore-formation [132].

Gallidermin and epidermin interfere with the biosynthesis of lipid-II, interact with lipid-I and II, and with their intermediates. Gallidermin significantly inhibits Staphylococcal biofilm formation by repression of *atl* (autolysin) and *ica* (inter-cellular adhesin) genes known to be involved in the formation of biofilms [50]. However, the antibiofilm activity of gallidermin was significantly decreased in mature biofilms (24 h–5 days old) [50].

Biosurfactants (BSs), also called microbial surfactants, are amphipathic (and surface-active) molecules formed by microorganisms, exhibit antimicrobial activity, and inhibit surface adhesion of bacterial cells, causing biofilm disruption. The potential antimicrobial, anti-adhesive, and dynamically active dispersal properties of biosurfactants made them promising antibiofilm compounds for biofilm eradication. Some effective biosurfactants are listed in Table 3. Sophorolipid BSs increase membrane permeability and disrupt the biofilm development of *B. subtilis*, *E. coli,* and *P. aeruginosa* in combination with caprylic acid [53]. *Lactobacillus casei*-produced BSs inhibited the biofilm formation of *S. aureus* [135].

### 4.3. Peptidoglycan Cleavage

The peptidoglycan layer, located in the cell walls of many bacteria, is formed from amino acids and sugars. The cleavage of peptidoglycan is also known to inhibit biofilm formation [143]. The peptidoglycan cleavage inhibits biofilm formation in several ways: it causes a change in protein composition, the amount of teichoic acid in the bacterial cell wall and can result in the release of signaling molecules modulating biofilm gene expression [143]. A peculiar group of peptidoglycan hydrolases encoded by bacteriophages is referred to as endolysin [86]. They are often species-specific, bind to the bacterial cell wall, and digest it, resulting in hypotonic cell lysis and bacterial cell death leading to progeny bacteriophage release [144]. Endolysin can work on multidrug-resistant strains, e.g., *PlyC* (specific *Streptococcal* bacteriophage) endolysin disrupts in vitro biofilms. Designing a bacteriophage-based treatment requires in-depth information of the infection-causing bacteria to properly design bacteriophages [145,146,147,148]. A polyphenol molecule, epigallocatechin gallate, binds with peptidoglycan and causes damage to bacterial cell walls leading to bacterial inhibition, which ultimately interferes with the primary or docking stage of biofilm formation [149,150,151].

The tannic acid (a polyphenolic compound) can inhibit the biofilm formation of *S. aureus* without inhibiting bacterial growth [85]. The mode of action of tannic acid depends upon the putative lytic transglycosylase (an immune-dominant Staphylococcal Antigen-A, IsaA), which causes peptidoglycan cleavage [152]. The transglycosylase acts like a lysozyme enzyme that induces the b-1,4 glycosidic bond cleavage between the N-acetyl glucosamine (NAG) and N-acetyl muramic acid (NAM) [153]. Tannic acid can inhibit biofilm formation by elevating the IsaA extracellular level [85].

### 4.4. Inhibition of Bacterial Cell Division

Cell division of bacteria is a vital process for the growth of bacterial biofilms. Cytoplasmic proteins play a part in cell division and further their survival. Few peptides having antimicrobial activity inhibit cytoplasmic proteins by penetrating the bacterial cytosol via a flip-flop of phospholipids or forming channels in an outer membrane. Some proline-rich antimicrobial peptides (AMPs) such as drosocin, pyrrhocoricin, and apidaecin have the potential to bind with a heat shock protein of bacteria (DnaK), impeding the initiation of cDNA (chromosomal DNA) replication [87,154]. They can also interfere with DnaJ (heat shock protein) and DnaK interactions causing bacterial death. AMPs with abundant proline can enter bacterial cells and bind to the ribosome tunnel, consequently interfering with protein synthesis [155].

### 4.5. Biofilm Dispersion

Biofilm disassembly is based on a series of steps that cause alteration in cellular physiology and extracellular matrix deterioration [91]. Most bacterial species can produce surfactants or extracellular enzymes that can degrade or solubilize the biofilm matrix [156]. If the matrix is removed, bacterial cells become separated from the biofilm and are released into the environment. Several biofilm matrix-degrading compounds, such as DNases, proteases, and surfactants, can mediate the active dispersion of biofilms [157].

In various bacteria, an accessory gene regulation (AGR) setup is present that controls the production of matrix deteriorating enzymes [158]. Auto-inducing peptide (AIP) has been demonstrated to mediate the AGR setup. Even at low (nanomolar) concentrations, AIP can activate a two-component signal transduction cascade system, which results in the production of virulence factors [159]. Several toxins (called phenol-soluble modulins) and some proteases are found in the extracellular proteome of the AGR setup [160]. AGR activation inhibits the maturation of biofilms [24]. The generation of extracellular proteases has also been linked to the disassembly of biofilms [160,161,162]. During the disassembly of biofilms, nuclease (effective DNase is also known as micrococcal nuclease and thermonuclease) works as an endogenous mediator that helps in bacterial cell separation from biofilm [163]. Restriction enzymes and DNases have been documented to cause biofilms dispersion [164]. Two bioactive molecules, parthenolide (sesquiterpene lactone) and AA-861 (a benzoquinone derivative), showed a significant inhibitory role against *Bacillus cereus, E. coli*, and *B. subtilis* biofilms by intervening with TasA polymerization into amyloid-like fibers [165].

Enterobacteriaceae species such as *E. coli* develop functional bacterial amyloids termed curli, involved in biofilm formation [166]. Curli accelerates the cell-cell or cell-surface contact to mediate the biofilm assembly to animate (plant and mammalian cells) as well as inanimate surfaces (plastic, glass, and stainless steel) [167]. Cegelski et al. reported attenuation of bacterial virulence in the urinary tract of an *E. coli* infected mouse model after pretreatment with FN075 [168]. Proteases and cysteine protease SpeB from *P. aeruginosa* and a Group A Streptococcus have been reported to initiate dispersal of bacterial biofilms, respectively [66,169].

D-tyrosine can prevent the attachment of bacterial cells, thus inhibiting biofilm formation. Moreover, at low concentration, it is able to initiate *P. aeruginosa* and *B. subtilis* biofilm disassembly. The D-tyrosine effect on extracellular protein and EPS-production in Gram-positive and negative bacteria is dosage-dependent [90]. Studies indicated that D-tryptophan, D-histidine, and D-cysteine could reduce *A. baumannii* biofilm formation by up to 35–86% at 2 mM concentration [90]. Moreover, D-tyrosine, D-cysteine, and D-tryptophan inhibited *P. aeruginosa* biofilm formation by up to 10–30% at 4 mM concentration. Recently, Bhoopalan et al. suggested that *nagZ* protein (involved in recycling peptidoglycan) can be used to disperse biofilms against the established *Neisseria gonorrhoeae* biofilm, but the mechanism of action is not yet clear [170].

### 4.6. Biofilm Inhibition via Polysaccharides

Extracellular polysaccharides (EPSs) are known to be essential elements of many biofilms [73]. Recently, it has been demonstrated that some polysaccharides can have detrimental effects on biofilm formation. EPSs have not only been shown to inhibit biofilm development, but they can also disturb the existing biofilm matrix [73,92]. An EPS named EPS-273 obtained from *P. stutzeri* 273 (a marine bacterium) inhibits biofilm development of *P. aeruginosa* by directly targeting the production of some virulence factors such as rhamnose, exo-protease, and pyocyanin. EPS-273 decreases pyocyanin production, resulting in a low amount of H_2_O_2_ production. In addition, it is also able to inhibit the eDNA release, which has been demonstrated to be an important factor in stable biofilm formation [171]. EPS-273 acts as an antioxidant and has been demonstrated to reduce biofilm-associated infections [94]. EPS-273 is effective against *P. aeruginosa* and could be used in healthcare settings to control nosocomial infections and in the food industry to prevent food spoilage [94].

Other antibiofilm polysaccharides have also been documented; Pel and Psl obtained from *P. aeruginosa* PAO1 decreased the formation of *S. epidermidis* biofilms [74,172]. PAM galactan and K2 polysaccharides obtained from *Kingella kingae* strain and *E. coli* capsules, respectively, can change biofilm structure by creating water channels and dispersing biofilms [173,174]. Exopolysaccharide A101 obtained from *Vibrio cholerae* QY101 can cause dispersion of *P. aeruginosa* biofilms [92]. Several polysaccharides besides those of bacterial origin obtained from algae, plants, and animals have been reported as antibiofilm molecules [73].

### 4.7. Bacterial Stringent Response Inhibition

Generally, (p)ppGpp (guanosine tetraphosphate and pentaphosphate) metabolism is regulated by two types of enzymes, RelQ and RelA. Previous studies revealed that RelA is responsible for regulating the accumulation of (p)ppGpp under stress conditions, whereas, with no stress lower expression of (p)ppGpp is regulated by RelQ. It was also indicated that alteration in (p)ppGpp could disturb biofilm structure and stability in vitro. The RelA enzyme catalyzes the bacterial stringent response and aids in the survival of bacterial cells during starvation by optimizing gene expression. Peptide 1018 inhibits the alarmone aggregation, a part of the bacterial stringent response, which is a result of nutritional stress [70]. Bacteria synthesize alarmones under stress conditions, collectively called (p)ppGpp [70,175]. The peptide 1018 causes damage to biofilms in three possible ways. Firstly, it inhibits or prevents biofilm formation when added before attachment; secondly, at very low concentrations, it disturbs biofilms and kills bacteria; thirdly, it can disrupt mature biofilms [70]. Synergistic effects with different antibiotics against bacterial biofilms have also been demonstrated [176]. Peptide 1018 and its two derivatives, HE4 and HE10, were effective against *B. cenocepacia* and *P. aeruginosa* even below their MIC. Peptide 1037 affects many Gram-negative and Gram-positive bacterial biofilms [95]. Peptide 1038 affects the twitching of bacteria and inhibits QS and bacterial adhesion of *Pseudomonas* cells. Eugenol, a secondary metabolite of *Syzigium aromaticum*, is able to induce gene downregulation during *S. mutans* treatment. Likewise, RelA is known to be involved in acid tolerance and in bacterial stringent response inhibition in biofilm formation [177].

### 4.8. Cyclic di-GMP System Signaling Inhibition

Bacterial communities can generally be categorized into three different types: (i) planktonic state that can cause acute bacterial infections but can generally be eliminated relatively quickly with antibiotics, (ii) biofilm state that is generally not easily treatable with antibiotics, and (iii) dispersed biofilms, a state that is defined as a shift between biofilm and planktonic states [178]. The dispersal process enables bacterial biofilms to proliferate within and between different hosts. The secondary messenger molecule cyclic di-GMP (c-di-GMP) is involved in biofilm development, and bacterial biofilm formation can be altered by modifying the c-di-GMP signaling pathway. During stress conditions, such as nitrosative and starvation conditions, microbial cells reduce the level of c-di-GMP via phosphodiesterase activation resulting in biofilm dispersal [178].

It has also been found that dispersed cells’ physiology and pathogenic capacity can be very different from planktonic cells and biofilms [179]. Dispersed biofilm cells can be highly virulent because of elevated virulent gene expression compared to planktonic and biofilm cells. Dispersed cells of biofilms exhibited a lower expression of rsmY and rsmZ along with the decreased concentration of c-di-GMP and caused less production of siderophores [179]. The siderophores chelate iron from the environment and appear to prevent biofilm formation by limiting biofilm dispersed cells’ survival. Chemical administration can also induce anti-biofilm activity by biofilm dispersal. The dispersed biofilm cells can escape from the macrophage conciliated phagocytosis process. Therefore, the addition of a few antimicrobials, along with different dispersing agents, is highly preferable to avoid the spread of dispersed cells and impede their growth [179].

Further, it is revealed that the addition of an iron-chelating agent with an antimicrobial and dispersing agent would feasibly eliminate several biofilms [179]. Some small molecules, including LP-1062, LP-3134, LP-3145, and LP-4010, obstruct the c-di-GMP production by inhibiting diguanylate cyclase (DGC), resulting in the formation inhibition of *A. baumannii* and *P. aeruginosa* biofilms. Though all of the above small molecules have been documented as effective *P. aeruginosa* biofilm dispersal inhibitors, only two of them were non-toxic toward eukaryotic cells at the concentrations used [180].

### 4.9. Enzymatic Dispersal of the Extracellular Polysaccharide Substance (EPS) of Biofilm

The EPS of biofilm matrix protects the microbes from different antimicrobial compounds, and EPS disassembly is known to aid in exposing bacterial cells to antimicrobials. Many enzymes, including DNases and polysaccharide lyases, are capable of degrading exopolysaccharides [36]. Dispersin-B and DNase-I enzymes, for instance, are known as significant anti-biofilm agents [181]. Dispersin-B is capable of glycosidic hydrolysis to cleave polymers, whereas DNase-I digests extracellular DNA often incorporated into EPS. These enzymes can be applied to disperse EPS layers found on medical instruments [164,182]. The bacterial biofilm-dispersing enzymes work more efficiently in killing EPS-embedded bacteria when used in combination with different antibacterial agents [183]. The EPS degradation decreases cellular attachment and drug resistance in microbial biofilms [184].

### 4.10. Lipopolysaccharide Disassembly or Neutralization

Antimicrobial peptides are antimicrobial agents known as an alternative to conventionally available antibiotics. AMPs are evolutionary-generated, conserved proteins that manifest antimicrobial activity against many bacterial, fungal, and viral pathogens [185]. Mostly, they are positively charged having both hydrophobic and hydrophilic moieties enabling them to dissolve in aqueous environments and penetrate the lipid bilayer of cells [186]. A lytic peptide, PTP-7, a synthetically obtained analog of Gaegurin 5, can penetrate deep inside the biofilm matrix and actively kill the bacteria [67]. Polymyxins, particularly polymyxins B and E, also called colistin, have been shown to bind with LPS and permeabilize the bacterial (Gram-negative) outer membrane [97,187]. A membrane-active AMP, Gramicidin S, is a broad-spectrum agent (active against both Gram-positive and negative bacteria) that actively disrupts the integrity of bacterial cell membranes [97,188].

## 5. Use of Natural Products

Ancient research reported herbs and spices aiding in food preservation and having medical benefits. Since the beginning of the 19th century, experimental studies have concluded that various natural compounds have antimicrobial properties [189]. However, the anti-biofilm activity of many of these compounds has not been validated in detail. Recently, various natural compounds with anti-biofilm properties, including plant extract, honey, and essential oils, have been studied in more detail.

### 5.1. Honey

Honey is likely the most widely used natural product with medicinal, antimicrobial, anti-inflammatory, antioxidant, and wound-healing properties. Significant antimicrobial activity against ~60 different fungal and bacterial species has been reported [190]. Maddocks et al. reported honey as a potent inhibitor of *Streptococcus pyogenes* biofilm formation. Honey was also observed to be an effective agent against biofilm development of *Enterococcus* spp., and may thus be employed as a therapeutic compound against biofilm-linked enterococcal infections [191,192]. Some authors revealed that honey could reduce virulence, QS, and biofilm formation of *E. coli* O157:H7 even at low concentrations. It was demonstrated that honey could effectively reduce biofilm formation of Enterohaemorrhagic *E. coli* (O157:H7) even at low concentrations by inhibiting QS and bacterial virulence genes without causing inhibition to cell growth. Honey can inhibit bacterial adhesion and biofilm formation at high concentrations owing to its strong antibacterial properties [193]. Besides honey’s antimicrobial properties, it can also inhibit biofilm formation due to an antimicrobial peptide, bee defensin-1, inhibiting microbial viability [194]. An in-depth study of honey’s mechanisms of action involved in biofilm inhibition and prevention is still under consideration [195]. These findings can primarily be used to establish a cost-effective natural anti-biofilm agent without causing any harmful side effects.

### 5.2. Plant Extracts

The anti-biofilm outcomes of natural compounds principally depend on the following features, decline in the production of virulence factors, inhibition of polymer matrix formation, suppression of cellular adherence, and consequently disrupting QS and biofilm formation. Some of the anti-biofilm agents extracted from natural products along with their target organisms and anti-biofilm effects are listed in Table 4. Plant extracts and their active components have been explored to eliminate microbial biofilms. Various plant extracts, including *Rhodiola crenulata*, *Epimedium brevicornum*, *Dolichos lablab*, *Polygonum cuspidatum,* and *Malus pumila,* have demonstrated anti-biofilm activity. Extracts of *E. brevicornum* and *P. cuspidatum* and their most active components, resveratrol and icariin, showed potent anti-biofilm activity, even when applied below their MIC concentrations [196,197].

*Melia dubia* extracts exhibited cogent suppression of hydrophobicity, bacterial swarming motility, hemolysis, and *E. coli* biofilm development at a concentration of 30 mg/mL [198]. Zuo et al. studied a combination therapy using coumarins (imperatorin, isoimperatorin, and coumarin) combined with antibiotics (ceftazidime and ampicillin). Coumarin effectively inhibited *P. aeruginosa* biofilms, and the antibiotic efficacy was synergistically enhanced using antibiotics in combination with coumarins [199].

Caper bush extract (*Capparis spinosa*) showed significant inhibition of EPS-production and biofilm development in *Proteus mirabilis*, *Serratia. marcescens*, *P. aeruginosa*, *Chromobacterium violaceum,* and *E. coli* biofilms. A concentration of 2 mg/mL resulted in the dispersion of established biofilms of these species [58]. A flavanone glycoside (naringin) extracted from grape and citrus fruits showed significant treatment efficacy against *P. aeruginosa* biofilms compared to the conventionally available antibiotics tetracycline and ciprofloxacin [200].

**Table 4:** Some natural products as anti-biofilm molecules along with their target organisms and antibiofilm effects.

Plant Extracts	Target Organisms	Anti-Biofilm Effects	References
*Bergenia crassifolia*	*S. mutans*	Reduced the adherence of *S. mutans* by inhibiting glucosyltransferases	[201]
Erianin	*S. aureus*	Inhibited cell adherence by down-regulating Sortase A	[202]
Hordenine	*P. aeruginosa*	Obstructed QS-linked phenotypes to decrease virulence factors and biofilm development	[203]
*Hymenocallis littoralis*	*C. albicans,* *S. aureus*	Antimicrobial, anti-biofilm, and antioxidant activities	[204]
Parthenolide	*P. aeruginosa PAO1*	Inhibited QS-linked gene expression (LasR, Lasl, RhlR and RhlI) and induced extracellular polymeric substance downregulation	[205]
Patriniae	*P. aeruginosa*	Reduced EPS synthesis and inhibited biofilm formation	[206]
Phloretin	*S. aureus SA1199B and RN4220*	Anti-biofilm formation	[207]
Quercetin	*S. pneumoniae*	Blocked Sortase A functioning, sialic acid synthesis, and impaired *S. pneumoniae* biofilm formation	[208]

Fresh extract of *Allium sativum* showed potent inhibition of *P. aeruginosa* biofilms reducing up to 6 log units [55]. Fruit extracts of *Bauhinia acuruana* and *Chamaecrista desvauxii*, leaf extracts of *Pityrocarpa moniliformis,* and branch extracts of *B. acuruana* successfully inhibited biofilm formation [209]. Dandasa (*Juglans regia*) and green tea also showed significant antibiofilm activity at concentrations of 6.2 and 12.5 mg/mL, respectively, against *E. coli* [210].

Abidi et al. examined the antibiofilm effect of five different plant extracts, including neem (*Azadirachta indica*), sea buckthorn (*Hippophae rhamnoides*), dandasa (*Juglans regia*), cranberry (*Vaccinium oxycocos*), and culinary spices against *M. smegmatis* biofilms [56]. *A. indica* extract was found to be the most effective antibiofilm against *M. smegmatis* biofilm [56]. This finding can be used to establish effective antibiofilm against other biofilm-producing *Mycobacteria*. Fruit extracts of a Southeast Asian medicinal plant, *Lagerstroemia speciosa,* exhibited significant inhibition of biofilm formation at 10 mg/mL concentration [211].

Carneiro et al. tested biofilm inhibition by *Casbane diterpene* extracts from the Brazilian plant *Croton nepetaefolius*. The anti-biofilm potential of *casbane diterpenes* was evaluated against Gram-negative (*P. fluorescence*, *P. aeruginosa*, *K. pneumoniae, E. coli*, and *K. oxytoca*) and Gram-positive bacteria (*S. aureus* and *S. epidermidis*) as well as yeast species (*C. albicans*, *C. tropicalis,* and *C. glabrata*). The authors reported the most significant biofilm inhibition against Gram-positive and one Gram-negative bacteria such as *P. aeruginosa* [59].

### 5.3. Essential Oils

Essential oils (EOs) are volatile substances extracted from natural plants, which have been used extensively against several pathogens. EOs are also used in the food industry due to their antimicrobial and preservative properties. EOs specifically cause damage to microbial cell walls, and it has been reported that different EOs inactivates microbes without evolving antimicrobial resistance [212]. Remarkably, their quick and easy degradation, low toxicity, and availability of a wide variety of EOs make them reliable natural anti-biofilm agents [213]. Cinnamon oil, widely used in the food industry, has effectively inhibited the biofilm development of *Lactobacillus plantarum*, *S. mutans,* and *S. epidermidis* [214]. EO of *Cinnamomum cassia* was analyzed for its antimicrobial effect against single or mixed species biofilms of *Enteropathogenic E. coli* (EPEC) and *L. monocytogenes* grown on stainless steel coupons [215].

Cumin oil is also a popular EO derived from *Cuminum cyminum*. It belongs to an aromatic medicinal plant of the family Apiaceae that is widely used in different medical formulations and the food industry. Cumin seeds have been traditionally used in different medicines for hundreds of years. Cumin oil has been used for the treatment of cough and bronchopulmonary disorders (as an astringent), digestive system disorders (as a eupeptic and carminative), etc. [216]. Safoura et al. examined the effectiveness of cumin seed oil against *K. pneumoniae* biofilms and observed the enhanced efficacy of ciprofloxacin in combination with cumin oil and reduced biofilm formation [217].

Oregano oil inhibited the biofilm formation of *S. aureus*, *S. sciuri*, *S. haemolyticus*, and *S. lugdunensis* at low concentrations [218,219]. Tea tree essential oil (TTO) has effective antibacterial activity, but its combination with conventional ciprofloxacin significantly enhanced the activity against *P. aeruginosa* biofilm formation. The results indicated the synergistic effect of CIP and TTO, which reduced the biomass of *P. aeruginosa* biofilms by 70% [220]. Szczepanski and Lipski reported the effectiveness of cinnamon, oregano, and thymol essential oils against three biofilm-producing bacterial strains (*Stenotrophomonas*, *Acinetobacter,* and *Sphingomonas*) [221]. It was observed that among these three essential oils, two of them exhibited inhibitory effects on the formation of biofilm at the minimum inhibitory concentration [222]. Thyme oil was the most significant biofilm formation inhibitor, even at 0.001% (*w*/*v*) concentration [215]. Filogonio et al. analyzed the anti-biofilm effect on commercially available dentifrice to control dental biofilms. These researches indicated that merging vegetable EO with commercially available dentifrice enhanced the dental-related biofilm control that may facilitate in the cure or treatment of periodontal diseases and dental caries [223].

## 6. Antibodies as a Combating Strategy

Recently, antibodies (Abs) have also been considered as antibiofilm agents for biofilm eradication. Monoclonal Abs (mAbs) have been found to bind with Psl, an EPS of *P. aeruginosa*, preventing biofilm formation. Psl facilitates the colonization of host tissues by *P. aeruginosa* [224]. The mAbs inhibited attachment of *P. aeruginosa* to host cells and provided considerable protection in different *P. aeruginosa* infected animal models, including a mouse model with thermal injury and acute-lethal pneumoniae [225]. *S. aureus* and *S. epidermidis* generate the surface polymers Poly-N-acetyl-β(1-6) glucosamine (PNAG) that facilitates biofilm formation. Human neutrophil origin Abs has killing effect on de-N-acetylated PNAG exhibiting *S. aureus* strains. Likewise, the passive immunization of mice using anti-dPNAG conjugated with DT (diphtheria toxoid) rabbit sera significantly increased the killing of *S. aureus* [226].

## 7. Nanotechnology-Based Combating Strategies

**Figure 6:**
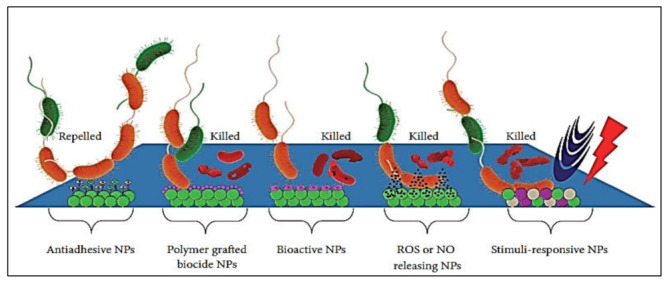
Anti-biofilm activity of surface engineered NPs with different antimicrobial effects (adapted from Lee et al. [244]).

Nanotechnology-based strategies offer propitious advances to inhibit biofilms and biofilm-associated infections by designing multi-targeted treatment avenues. Combining nanotechnology with chemical engineering methods provides versatility for optimizing the composition, shape, size, surface, and functional chemistry of nanomaterials (NMs) with the construction of modified material to prevent biofilm formation [227]. Materials at the nanoscale level manifest exclusive biological and physicochemical properties, and comparatively, their bulk counterparts do not possess these properties [228]. The chemical and bioactivities of NMs are enhanced due to their high surface area. Functionalized NMs have been designed to increase microbial cell wall penetration, target site selection, and control drug release. Furthermore, NMs have greater plasma half-lives and large surface-area-to-volume ratios that mediate drug loading and selective targeting [229]. Only a few studies are available that describe the use of nanoparticles (NPs) as surface coatings to inhibit biofilm formation [230]. Recent findings in the field of nanotechnology established unique possibilities for significant biofilm killing and control. The mode of action of NPs is believed to act by forming free radicals or producing oxidative stress to damage DNA. Modes responsible for the antimicrobial activity of different NPs may comprise certain properties such as composition, size [231], surface charge [232], and shape [233]. NPs are described to be involved [234,235,236,237] in membrane alterations, ROS generation [238], dropping of respiratory activity [239], the unwinding of DNA [240], metabolic pathway disruption [241,242], and lipid peroxidation [243]. A brief overview of certain surface-modified NPs to allow for the control or prevention of biofilms on biomedical devices with their relevant mode of action is presented in Figure 6. The NPs include polymeric NPs, metal NPs, metal-polymer nanocomposites, NO/ROS releasing NPs, stimuli-responsive NPs, and bioactive NPs.

NPs are an alternative to traditional antibiotic therapy to combat biofilm-associated and multi-drug-resistant infections [237]. Different types of NPs have been designed as antimicrobial and antibiofilm agents, such as organic, inorganic, metal, and green NPs, as well as combinations of them (Table 5) [245]. Silver (Ag) is a potent antimicrobial, and AgNPs have been used in different disinfectants. AgNPs exhibit several antimicrobial actions, including adhesion to and penetration into microbial cells resulting in increased membrane permeability and cell disintegration [246].

An investigation has revealed strong antibiofilm activity against five biofilm-producing multi-drug resistant bacteria (*E. coli*, *A. baumannii*, *P. mirabilis, K. pneumoniae*, and *P. aeruginosa*) using AgNPs [251]. Poly-L-lysin entrapped rifampicin, encapsulated in poly-lactic acid (PLA) nanoparticles prepared through nanoprecipitation, effectively enhanced the retention time and antibiotic efficacy against *S. aureus* biofilms [252].

Biologically synthesized AgNPs using *β*-1,3 glucan binding protein have shown 80% and 85% inhibition against immature biofilms of *P. aeruginosa* and *E. faecalis*, respectively [253]. AgNPs synthesized using a medicinal plant, *Crataeva nurvala*, significantly repressed the synthesis of QS-mediated virulence factors, including hemolysin, pyocyanin, and protease, and inhibited biofilm development of *P. aeruginosa* [254]. NPs offer a promising therapeutic approach for effective biofilm targeting, and the design of novel NPs is continuing. However, there remains a gap between the different formulations under lab examination and their successful clinical use. Future research and development activities should be focused on improved biocompatibility, metabolism, reduced toxicity, and enhanced in vivo effectiveness of NPs inside the body. Cost-effective large-scale production would also be needed for product manufacturing at the commercial level.

## 8. Anti-Biofilm Compounds with Unknown Mode of Action

**Figure 7:**
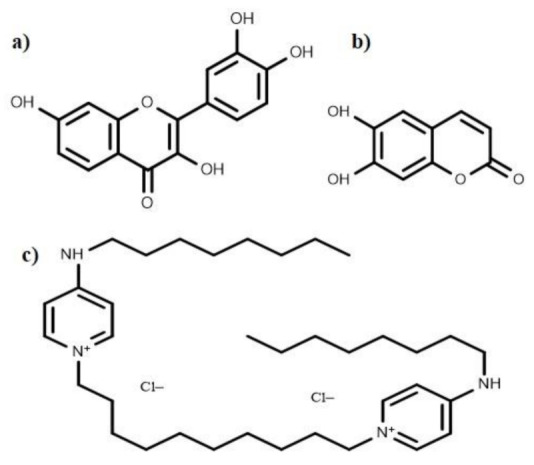
Chemical structures of the anti-biofilm compounds with unknown mode of action; (**a**) fisetin, (**b**) esculetin, (**c**) octenidine hydrochloride.

Several anti-biofilm compounds have been found to work very effectively against various bacteria, but their mode of action remains unknown. Secondary metabolites such as esculetin and fisetin (Figure 7) efficiently inhibit biofilm formation [60]. Esculetin activity prevents the maturation of biofilms resulting in thinner biofilms [60]. Fisetin treatment not only decreases the thickness of biofilms; it also hinders with the onset of bacterial biofilm formation by inhibiting biofilm regulatory protein (BrpA). Bispyridinamine, a positively charged octenidine hydrochloride, is also reported to be a significant anti-biofilm molecule, but its mechanism of action is yet to be discovered. Studies revealed that this molecule could be used as an antimicrobial lock solution and sanitizer in prophylactic and treatment activities [255].

## 9. Cytotoxicity Assessment Methods

Cytotoxicity is one of the most important properties for evaluating any deleterious effects of anti-biofilm agents before their commercialization for controlling and eliminating biofilms. There are several methods available for the evaluation of cytotoxic effects such as the MTT assay (metabolic activity assessment, colorimetric assay) [256,257], Trypan blue (living cell exclusion) [258], crystal violet [259], LDH assay (lactate dehydrogenase activity assessment) [260], XTT assay (used to quantify cellular viability, proliferation and cytotoxicity) [261], colony formation technique [259], PI (propidium iodide) [262], DAPI (4′,6-diamidino-2-phenylindole) staining, and others [263]. Some ISO (international standard organization) standards (such as ISO-22196) have been developed to evaluate the antibacterial and anti-biofilm properties of antimicrobial agents [264]. Anti-biofilm efficacy of antimicrobial agents can also be determined using the American Standards for Testing and Materials (ASTM) protocols [265]. The five different protocols (ASTM E2871, ASTM E2799, ASTM E2562, ASTM E2647, and ASTM E2196) developed by ASTM are available for standardized evaluation [266,267]. Natural plant-derived anti-biofilm agents are often non-toxic or less toxic. Cytotoxicity studies have been conducted using various bacterial species, and their results indicated that the gastrointestinal tract and mucous membrane do not absorb octenidine hydrochloride with any reported mutagenicity, carcinogenicity, or genotoxicity [268].

It has been found that usnic acid can cause allergic side effects such as local irritation and contact dermatitis. During in vitro studies, when this compound was tested separately or as a part of an oral formulation, no adverse cytotoxic effects became evident [64,65]. Various tests in drosophila infection models revealed that the anti-biofilm compound S-phenyl L-cysteine sulfoxide and its derivative (diphenyl disulfide) do not possess any lethal and toxic effects [269]. Similarly, different antibiofilm compounds, such as AMPs, have no cytotoxic effects [270]. In addition to cytotoxicity assessments of antibiofilm compounds, other considerations are also essential. Plasma protein binding, solubility, permeability, and efflux studies are necessary before more certainty in the safety and efficacy of anti-biofilm compounds can be obtained.

## 10. Obstacles in the Development of Therapeutic Strategies

The biofilm formation process encompasses dynamic and intricate interactions among microbes, EPS, and the surface. The visco-elastic properties and the adhesion strength of microbes facilitates biofilm formation and makes them resistant to antimicrobial compounds. A crucial challenge in biofilms treatment is that the use of antimicrobial substances alone often leaves microscopic residues of biofilms and cell debris; while cells might survive in the microscopic residues, the residues will also likely facilitate colonization by microbes in the future. For instance, previously dispersed cells could become virulent again once treatment has stopped or after becoming resistant. Based on the above outcomes, it has been concluded that the treatment strategy should target both EPS and the residual microorganisms.

Furthermore, the capability of an antimicrobial to penetrate established biofilms might also be essential. This aspect may induce the development of antimicrobial resistance (de novo) due to microbes being exposed to sub-lethal doses of antibiotics and potential cytotoxicity effects [179,271]. One significant strategy may be to target the immediate environment of pathogens to create hypoxia, extreme pH, and potentially pathogen-originated metabolites to generate the dispersal of biofilms. Using this strategy, the biofilm matrix can be degraded, and the resident microorganisms can be killed, resulting in pathogenic niche eradication with less cytotoxicity. In vivo scrutinization and clinical investigations exploring efficient dispersive agents to eliminate pathogenic biofilms are still limited. Most in vitro studies have been conducted using mono-species biofilms, and it remains challenging to perform these studies reliably using multi-species biofilms.

Moreover, the effect of treatments on the host (e.g., cytotoxicity) or vice versa (e.g., degradation or inhibition of therapeutics by the host) must be kept in mind [272,273]. The challenges discussed in this and the previous sections explain some of the challenges in commercializing biofilm treatment suitable for clinical treatments. However, the development of effective biofilm treatments is essential, and despite the outlined challenges, it remains a promising and mesmerizing research field. Combinatorial and multitarget strategies are emerging as a promising avenue that may prove to be key in effectively treating microbial biofilm infections in the future.

## 11. Conclusions and Future Perspectives

The emanation of perilous biofilm-associated infections and the emergence of antimicrobial resistance are formidable challenges globally. Biofilm formation on medical devices, surfaces, and food products is a major challenge for health systems worldwide. Conventional therapies are often limited in their efficacy in inhibiting microbial biofilms and combating strategies that rely on disinfectants and broad-spectrum antibiotics. Due to the high resistance of microbes to antibiotics, the development and application of highly effective anti-biofilm treatments have become crucial to successfully managing biofilm-associated infections. Biofilms are known to cause several harmful infections such as dental diseases, middle ear infections, urinary tract infections, catheter-associated infections, bacterial vaginosis, skin ulcers, prosthetic joint and oral implant infections, contact lenses, and orthopedic implant infections.

Moreover, some infections are not as prevalent and noticeable but can be more pernicious such as endocarditis and cystic fibrosis. Therefore, there is an essential need to establish effective biofilm combating strategies. This review summarized and scrutinized information on various biofilm combating strategies and modes of action of different anti-biofilm agents. It is concluded that revamping or modifying currently available drugs may be a productive idea, as well as evaluating combination treatments on medically relevant biofilms, in vitro and in vivo. Numerous bioinformatics tools can be applied to screen existing antibiofilm agents and their remodeling. In the future, it is expected that vaccinations can be used as an effective biofilm combating approach. Potential proteins or antigens and their target sites are some of the obstacles in vaccine design and development, due to the diversity of microbial proteins and antigens. Vaccine use can be combined with different antimicrobial, antibiotic, and combination treatments.

## Figures and Tables

**Table 1:** Some anti-biofilm compounds, their source, and a list of bacteria for which these compounds have demonstrated treatment efficacy.

Antibiofilm Molecule		Source of Molecule	Susceptible Microorganism	References
Antibiotics and Lantibiotics	Epidermin	*Staphylococcus epidermidis* (Tu3298)	*Lactococcus lactis*	[49,50,51,52]
	Gallidermin	*Staphylococcus gallinarum* (Tu3928)	*S. epidermidis* *S. aureus*	
	Nisin	*L. lactis*	*S. aureus* *S. epidermidis*	
	Polymyxin B	*Paenibacillus polymyxa*	*Escherichia coli* *S. aureus* *P. aeruginosa*	
	Polymyxin E (Colistin)	*P. polymyxa*	*Stenotrophomonas maltophilia*	
	Subtilin	*Bacillus subtilis* (ATCC6633)	*L. lactis*	
Biosurfactant	Sophorolipid	Produced on microbial cells	*S. aureus, B. subtilis* *Cupriavidus necator*	[53]
Chelating agents	Disodium-EDTA, Sodium citrate, Tetrasodium EDTA	-	*P. aeruginosa* *Staphylococcus* species	[54]
Enzymes	Deoxyribo-nuclease I, Glycoside hydrolase	-	*Staphylococcus* *Enterococcus*	[47,54]
Naturally derived and some other molecules	*Allium sativum*	Extract	*P. aeruginosa*	[55]
	*Azadirachta indica*	Plant extract	*Mycobacterium smegmatis*	[56]
	Berberine	*Berberis aquifolium*, *B. vulgaris*, *B. aristata*	*K. pneumoniae*	[57]
	Capparis spinosa	Caper bush extract	*Proteus mirabilis*, *P. aeruginosa*, *E. coli*, *Serratia marcescens*	[58]
	Casbane diterpene	*Croton nepetaefolius* extract	*P. aeruginosa*, *P. fluorescence*, *S. aureus*, *E. coli*, *K. pneumoniae*, *K. oxytoca*, *S. epidermidis*, *Candida albicans*, *C. tropicalis*, *C. glabrata*	[59]
	Curcumin	*Curcuma longa*	*K. pneumoniae*	[57]
	Ellagic acid	*Camellia sinesis*	*Streptococcus dysgalactiae*	[60]
	Epigallocatechin gallate (EGCG)	*Camellia sinesis* (Green tea)	*Acinetobacter baumannii*, *S. aureus*, *E. coli* *P. aeruginosa*	[51]
	Esculetin	*Alchemilla speciose,* *Santolina oblongifolia,* *Tagetes lucida*	*S. aureus*	[60]
	Eugenol	*Ocimum* plants, *Syzigium aromaticum*	*K. pneumoniae*, *Streptococcus mutans*	[57,61]
	Fiestin	*Allium cepa,* *Cucumis sativus,* *Fragaria ananassa,* *Malus domestica,* *Solanum lycopersicum,* *Vitis vinifera*	*S. aureus,* *S. dysgalactiae*	[60]
	Quercetin	*Usnea longissimi*	*P. aeruginosa,* *K. pneumoniae*	[62]
	Reserpine	*Rauwolfia vomitoria,* *R. serpentine*	*K. pneumoniae*	[57]
	Synthetic halogenated furanone (F-56)	Derived from natural furanone	*P. aeruginosa,* *Serratia liquifaciens*	[63]
	Usnic acid	*Secondary lichen metabolite*	*C. albicans,* *S. aureus*	[64,65]
Peptides	Antimicrobial peptide (AMP, LL-37)	Human cationic host defense peptide	*E. coli, S. aureus,* *P. aeruginosa*	[52,54,66,67,68,69,70,71,72]
	Buforin-II	Derived from stomach tissue (Buforin-I) of *Bufobufo gargarizans*	Gram-negative bacteria	
	Indolocidin	Isolated from bovine neutrophil-cytoplasmic granules		
	Lytic peptide (PTP-7)	Synthetic analog from *Gaegurin 5*		
	PMAP-23	*Cathelicdin*		
	PR-39	Pig’s small intestine		
	Sushi peptide	Factor C (sushi-3)		
	Microcin-B17	*E. coli* (Post-translationally modified peptide)	*E. coli*	
	Peptide 1018	-	*A. baumannii,* *B. cenocepacia,* *E. coli, S. aureus,* *P. aeruginosa,* *S. typhimurium*	[70]
Polysaccharides	CFT073 Group-II Capsular Polysaccharide	*E. coli*	*E. coli*, *S. aureus*, *K. pneumoniae,* *P. aeruginosa*	[73,74,75]
	Pel Polysaccharide, Psl Polysaccharide	*P. aeruginosa*	*S. aureus*	
Metallic nanocomposites	Zn-CuO	Chemical synthesis	*P. aeruginosa,* *S. epidermidis*	[76]
Inorganic NPs	Ag NPs Au NPs	Chemical synthesis *Capsicum annuum*	*S. aureus,* *P. aeruginosa*	[77,78]
Organic NPs	Quaternary ammonium chitosan NPs, PEG stabilized lipid NPs	Chemical synthesis	*Candida albicans*	[79]

**Table 2:** Modes of actions followed by several anti-biofilms.

Serial. No.	Mode of Action	Associated Agents	References
1	AHL-mediated QS inhibition	Halogenated furanones, Flavonoids (quercetin)	[80,81]
2	Membrane permeabilization or potential alteration	Lytic peptides (PTP-7), Lantibiotics (gallidermin, nisin), Biosurfactants (sophorolipids), Organic NPs (e.g., Quaternary ammonium chitosan NPs, PEG stabilized lipid NPs)	[79,82,83,84]
3	Peptidoglycan cleavage	Epigallocatechin gallate (EGCG), Tannic acid, Endolysins	[85,86]
4	Inhibition of bacterial cell division and their survival	Microcin-B17, Pyrrhocoricin	[87,88]
5	Bacterial inhibition via biofilm disassembly	Extracellular proteases (Esp, sarA, sigB), D-tyrosine, Nucleases, Anti-amyloids, A cyclic auto inducing peptide (AIP), Ethyl pyruvate	[89,90,91]
6	Biofilm inhibition via polysaccharides	Pel and Psl, PAM galactan, ESP-273, K2 Polysaccharides	[73,92,93,94]
7	Bacterial stringent response inhibition	Peptide-1018, Peptide-1038	[70,95]
8	Cyclic di-GMP System signaling inhibition	LP-1062, LP-3134, LP-3145, LP-4010	[4]
9	Enzymatic dispersal of the extracellular polysaccharide substance (EPS) of matrix biofilm	Dispersin-B, DNase-I, Inorganic NPs (e.g., Ag NPs, Au NPs)	[77,78,96]
10	Lipopolysaccharide disassembly or neutralization	Polymyxin-B and E, Lytic peptide, Gramicidin-S	[97]

**Table 3:** Biosurfactants inhibiting biofilm formation.

Biosurfactant	Source	Effective Against	References
Coryxin	*Corynebacterium xerosis*	*S. mutans*, *E. coli*, *S. aureus* and *P. aeruginosa*	[136]
Pontifactin	*Pontibacter korlensis*	*S. aureus*, *V. cholera, Salmonella typhi* and *B. subtilis*	[137]
Rhamnolipid	*Burkholderia thailandensis,* *P. aeruginosa*	*Neisseria mucosa*, *Streptococcus orails*, *Streptococcus sanguinis, Actinomyces naeslundii*	[138,139]
Sophorolipid	*Candida bombicola*	*S. aureus* and *B. subtilis*	[53]
Surfactin, iturin, and fengycin	*B. subtilis*	Biofilm formation of uropathogenic bacteria	[140]
NS	*Acinetobacter indicus*	Treatment of seven days old biofilms	[141]
NS	*Lactobacillus gasseri and Lactobacillus jenesenii*	*E. coli*, *Enterobacter aerogenes*, *Staphylococcus* and *Saprophyticus*	[142]

NS-Not specified.

**Table 5:** Nanotechnology-based materials for the control and treatment of biofilm-associated infections.

Nanomaterials		Mode of Action	Antibiofilm Devices	References
Organic NPs	PEG stabilized lipid NPs, Quaternary ammonium chitosan NPs	Disrupt the biofilms by inducing ion-exchange via penetrating the cell membrane	Bones, Dental cements	[79]
Inorganic NPs	Au NPs Ag NPs	Positive surface-charge damages the EPS network. Interaction of Ag ions with bacterial sulfhydryl groups disrupts the integrity of cell membranes, enzymatic activities, cell proliferation, etc.	Catheters	[247,248]
Metallic nanocomposites	Zn-CuO nanocoating, Ti-implant surfaces with ZnO NPs	Released Ag ions inhibit biofilm formation. Direct contact	Contact lenses, Dental implants	[249,250]

## Data Availability

Not applicable.

## References

[1] Percival S.L., Malic S., Cruz H., Williams D.W. (2011). Introduction to biofilms. Biofilms and Veterinary Medicine.

[2] Stewart P.S. (2002). Mechanisms of antibiotic resistance in bacterial biofilms. Int. J. Med. Microbiol..

[3] Davies D. (2003). Understanding biofilm resistance to antibacterial agents. Nat. Rev. Drug Discov..

[4] Wu H., Moser C., Wang H.-Z., Høiby N., Song Z.-J. (2015). Strategies for combating bacterial biofilm infections. Int. J. Oral Sci..

[5] Piozzi A., Francolini I., Occhiaperti L., Di Rosa R., Ruggeri V., Donelli G. (2004). Polyurethanes loaded with antibiotics: Influence of polymer-antibiotic interactions on in vitro activity against *Staphylococcus epidermidis*. J. Chemother..

[6] Donelli G., Francolini I. (2001). Efficacy of antiadhesive, antibiotic and antiseptic coatings in preventing catheter-related infections. J. Chemother..

[7] Hengzhuang W., Wu H., Ciofu O., Song Z., Høiby N. (2011). Pharmacokinetics/Pharmacodynamics of Colistin and Imipenem on mucoid and non-mucoid *Pseudomonas aeruginosa* biofilm. Antimicrob. Agents Chemother..

[8] Hengzhuang W., Wu H., Ciofu O., Song Z., Høiby N. (2012). In vivo pharmacokinetics/pharmacodynamics of colistin and imipenem on biofilm *Pseudomonas aeruginosa*. Antimicrob. Agents Chemother..

[9] Høiby N., Ciofu O., Johansen H.K., Song Z.J., Moser C., Jensen P.Ø., Molin S., Givskov M., Tolker-Nielsen T., Bjarnsholt T. (2011). The clinical impact of bacterial biofilms. Int. J. Oral Sci..

[10] Cramton S.E., Gerke C., Schnell N.F., Nichols W.W., Götz F. (1999). The intercellular adhesion (ica) locus is present in *Staphylococcus aureus* and is required for biofilm formation. Infect. Immun..

[11] McKenney D., Hübner J., Muller E., Wang Y., Goldmann D.A., Pier G.B. (1998). The ica locus of *Staphylococcus epidermidis* encodes production of the capsular polysaccharide/adhesin. Infect. Immun..

[12] Aaron S.D., Ferris W., Ramotar K., Vandemheen K., Chan F., Saginur R. (2002). Single and combination antibiotic susceptibilities of planktonic, adherent, and biofilm-grown *Pseudomonas aeruginosa* isolates cultured from sputa of adults with cystic fibrosis. J. Clin. Microbiol..

[13] Rasmussen T.B., Givskov M. (2006). Quorum sensing inhibitors: A bargain of effects. Microbiology.

[14] Stewart P.S. (2015). Antimicrobial tolerance in biofilms. Microb. Biofilms.

[15] Ciofu O., Mandsberg L.F., Wang H., Høiby N. (2012). Phenotypes selected during chronic lung infection in cystic fibrosis patients: Implications for the treatment of *Pseudomonas aeruginosa* biofilm infections. FEMS Immunol. Med. Microbiol..

[16] Anderl J.N., Zahller J., Roe F., Stewart P.S. (2003). Role of nutrient limitation and stationary-phase existence in *Klebsiella pneumoniae* biofilm resistance to ampicillin and ciprofloxacin. Antimicrob. Agents Chemother..

[17] Brown M.R., Allison D.G., Gilbert P. (1988). Resistance of bacterial biofilms to antibiotics a growth-rate related effect?. J. Antimicrob. Chemother..

[18] Walters M.C., Roe F., Bugnicourt A., Franklin M.J., Stewart P.S. (2003). Contributions of antibiotic penetration, oxygen limitation, and low metabolic activity to tolerance of *Pseudomonas aeruginosa* biofilms to ciprofloxacin and tobramycin. Antimicrob. Agents Chemother..

[19] Ma H., Bryers J.D. (2013). Non-invasive determination of conjugative transfer of plasmids bearing antibiotic-resistance genes in biofilm-bound bacteria: Effects of substrate loading and antibiotic selection. Appl. Microbiol. Biotechnol..

[20] Bjarnsholt T., Jensen P.Ø., Fiandaca M.J., Pedersen J., Hansen C.R., Andersen C.B., Pressler T., Givskov M., Høiby N. (2009). *Pseudomonas aeruginosa* biofilms in the respiratory tract of cystic fibrosis patients. Pediatric Pulmonol..

[21] Gjødsbøl K., Christensen J.J., Karlsmark T., Jørgensen B., Klein B.M., Krogfelt K.A. (2006). Multiple bacterial species reside in chronic wounds: A longitudinal study. Int. Wound J..

[22] Kirketerp-Møller K., Jensen P.Ø., Fazli M., Madsen K.G., Pedersen J., Moser C., Tolker-Nielsen T., Høiby N., Givskov M., Bjarnsholt T. (2008). Distribution, organization, and ecology of bacteria in chronic wounds. J. Clin. Microbiol..

[23] Homøe P., Bjarnsholt T., Wessman M., Sørensen H.C.F., Johansen H.K. (2009). Morphological evidence of biofilm formation in Greenlanders with chronic suppurative otitis media. Eur. Arch. Oto-Rhino-Laryngol..

[24] Boles B.R., Horswill A.R. (2008). Agr-mediated dispersal of *Staphylococcus aureus* biofilms. PLoS Pathog..

[25] Van Oss C., Good R., Chaudhury M. (1986). The role of van der Waals forces and hydrogen bonds in “hydrophobic interactions” between biopolymers and low energy surfaces. J. Colloid Interface Sci..

[26] Merino N., Toledo-Arana A., Vergara-Irigaray M., Valle J., Solano C., Calvo E., Lopez J.A., Foster T.J., Penadés J.R., Lasa I. (2009). Protein A-mediated multicellular behavior in *Staphylococcus aureus*. J. Bacteriol..

[27] Corrigan R.M., Rigby D., Handley P., Foster T.J. (2007). The role of *Staphylococcus aureus* surface protein SasG in adherence and biofilm formation. Microbiology.

[28] Conrady D.G., Brescia C.C., Horii K., Weiss A.A., Hassett D.J., Herr A.B. (2008). A zinc-dependent adhesion module is responsible for intercellular adhesion in staphylococcal biofilms. Proc. Natl. Acad. Sci. USA.

[29] O’Neill E., Pozzi C., Houston P., Humphreys H., Robinson D.A., Loughman A., Foster T.J., O’Gara J.P. (2008). A novel *Staphylococcus aureus* biofilm phenotype mediated by the fibronectin-binding proteins, FnBPA and FnBPB. J. Bacteriol..

[30] Martí M., Trotonda M.P., Tormo-Más M.Á., Vergara-Irigaray M., Cheung A.L., Lasa I., Penadés J.R. (2010). Extracellular proteases inhibit protein-dependent biofilm formation in *Staphylococcus aureus*. Microbes Infect..

[31] Trotonda M.P., Manna A.C., Cheung A.L., Lasa I., Penadés J.R. (2005). SarA positively controls bap-dependent biofilm formation in *Staphylococcus aureus*. J. Bacteriol..

[32] Overhage J., Campisano A., Bains M., Torfs E.C., Rehm B.H., Hancock R.E. (2008). Human host defense peptide LL-37 prevents bacterial biofilm formation. Infect. Immun..

[33] Fuqua W.C., Winans S.C., Greenberg E.P. (1994). Quorum sensing in bacteria: The LuxR-LuxI family of cell density-responsive transcriptional regulators. J. Bacteriol..

[34] Anderl J.N., Franklin M.J., Stewart P.S. (2000). Role of antibiotic penetration limitation in *Klebsiella pneumoniae* biofilm resistance to ampicillin and ciprofloxacin. Antimicrob. Agents Chemother..

[35] Zhang L., Liang E., Cheng Y., Mahmood T., Ge F., Zhou K., Bao M., Lv L., Li L., Yi J. (2020). Is combined medication with natural medicine a promising therapy for bacterial biofilm infection?. Biomed. Pharmacother..

[36] Stewart P.S. (2015). Prospects for anti-biofilm pharmaceuticals. Pharmaceuticals.

[37] Stewart P.S., Bjarnsholt T. (2020). Risk factors for chronic biofilm-related infection associated with implanted medical devices. Clin. Microbiol. Infect..

[38] Høiby N., Johansen H.K., Moser C., Song Z., Ciofu O., Kharazmi A. (2001). *Pseudomonas aeruginosa* and the in vitroand in vivo biofilm mode of growth. Microbes Infect..

[39] Olson M.E., Ceri H., Morck D.W., Buret A.G., Read R.R. (2002). Biofilm bacteria: Formation and comparative susceptibility to antibiotics. Can. J. Vet. Res..

[40] Herrmann G., Yang L., Wu H., Song Z., Wang H., Høiby N., Ulrich M., Molin S., Riethmüller J., Döring G. (2010). Colistin-tobramycin combinations are superior to monotherapy concerning the killing of biofilm *Pseudomonas aeruginosa*. J. Infect. Dis..

[41] Francolini I., Piozzi A., Donelli G. (2014). Efficacy evaluation of antimicrobial drug-releasing polymer matrices. Microbial Biofilms.

[42] Van Dyck K., Pinto R.M., Pully D., Van Dijck P. (2021). Microbial Interkingdom Biofilms and the Quest for Novel Therapeutic Strategies. Microorganisms.

[43] Donelli G., Francolini I., Ruggeri V., Guaglianone E., D’ilario L., Piozzi A. (2006). Pore formers promoted release of an antifungal drug from functionalized polyurethanes to inhibit *Candida* colonization. J. Appl. Microbiol..

[44] Donelli G., Francolini I., Piozzi A., Rosa R.D., Marconi W. (2002). New polymer-antibiotic systems to inhibit bacterial biofilm formation: A suitable approach to prevent central venous catheter-associated infections. J. Chemother..

[45] Antonelli M., De Pascale G., Ranieri V., Pelaia P., Tufano R., Piazza O., Zangrillo A., Ferrario A., De Gaetano A., Guaglianone E. (2012). Comparison of triple-lumen central venous catheters impregnated with silver nanoparticles (AgTive^®^) vs. conventional catheters in intensive care unit patients. J. Hosp. Infect..

[46] Crisante F., Taresco V., Donelli G., Vuotto C., Martinelli A., D’Ilario L., Pietrelli L., Francolini I., Piozzi A. (2015). Antioxidant hydroxytyrosol-based polyacrylate with antimicrobial and antiadhesive activity versus *Staphylococcus epidermidis*. Advances in Microbiology, Infectious Diseases and Public Health.

[47] Donelli G., Francolini I., Romoli D., Guaglianone E., Piozzi A., Ragunath C., Kaplan J. (2007). Synergistic activity of dispersin B and cefamandole nafate in inhibition of staphylococcal biofilm growth on polyurethanes. Antimicrob. Agents Chemother..

[48] Walsh D.J., Livinghouse T., Goeres D.M., Mettler M., Stewart P.S. (2019). Antimicrobial activity of naturally occurring phenols and derivatives against biofilm and planktonic bacteria. Front. Chem..

[49] Parisot J., Carey S., Breukink E., Chan W.C., Narbad A., Bonev B. (2008). Molecular mechanism of target recognition by subtilin, a class I lanthionine antibiotic. Antimicrob. Agents Chemother..

[50] Saising J., Dube L., Ziebandt A.-K., Voravuthikunchai S.P., Nega M., Götz F. (2012). Activity of gallidermin on *Staphylococcus aureus* and *Staphylococcus epidermidis* biofilms. Antimicrob. Agents Chemother..

[51] Vidigal P.G., Müsken M., Becker K.A., Häussler S., Wingender J., Steinmann E., Kehrmann J., Gulbins E., Buer J., Rath P.M. (2014). Effects of green tea compound epigallocatechin-3-gallate against *Stenotrophomonas maltophilia* infection and biofilm. PLoS ONE.

[52] Park S.-C., Park Y., Hahm K.-S. (2011). The role of antimicrobial peptides in preventing multidrug-resistant bacterial infections and biofilm formation. Int. J. Mol. Sci..

[53] De Rienzo M.A.D., Banat I.M., Dolman B., Winterburn J., Martin P.J. (2015). Sophorolipid biosurfactants: Possible uses as antibacterial and antibiofilm agent. New Biotechnol..

[54] Abdel-Aziz S.M., Aeron A. (2014). Bacterial biofilm: Dispersal and inhibition strategies. SAJ Bio-Technol..

[55] Harjai K., Kumar R., Singh S. (2010). Garlic blocks quorum sensing and attenuates the virulence of *Pseudomonas aeruginosa*. FEMS Immunol. Med. Microbiol..

[56] Abidi S.H., Ahmed K., Sherwani S.K., Bibi N., Kazmi S.U. (2014). Detection of *Mycobacterium smegmatis* biofilm and its control by natural agents. Int. J. Curr. Microbiol. App. Sci..

[57] Magesh H., Kumar A., Alam A., Sekar U. (2013). Identification of natural compounds which inhibit biofilm formation in clinical isolates of *Klebsiella pneumoniae*. Indian J. Exp. Biol..

[58] Abraham S.V.P.I., Palani A., Ramaswamy B.R., Shunmugiah K.P., Arumugam V.R. (2011). Antiquorum sensing and antibiofilm potential of *Capparis spinosa*. Arch. Med. Res..

[59] Carneiro V.A., Santos H.S.d., Arruda F.V.S., Bandeira P.N., Albuquerque M.R.J.R., Pereira M.O., Henriques M., Cavada B.S., Teixeira E.H. (2010). Casbane diterpene as a promising natural antimicrobial agent against biofilm-associated infections. Molecules.

[60] Dürig A., Kouskoumvekaki I., Vejborg R.M., Klemm P. (2010). Chemoinformatics-assisted development of new anti-biofilm compounds. Appl. Microbiol. Biotechnol..

[61] Adil M., Singh K., Verma P.K., Khan A.U. (2014). Eugenol-induced suppression of biofilm-forming genes in *Streptococcus mutans*: An approach to inhibit biofilms. J. Glob. Antimicrob. Resist..

[62] Gopu V., Meena C.K., Shetty P.H. (2015). Quercetin influences quorum sensing in food borne bacteria: In-vitro and in-silico evidence. PLoS ONE.

[63] Hentzer M., Riedel K., Rasmussen T.B., Heydorn A., Andersen J.B., Parsek M.R., Rice S.A., Eberl L., Molin S., Høiby N. (2002). Inhibition of quorum sensing in *Pseudomonas aeruginosa* biofilm bacteria by a halogenated furanone compound. Microbiology.

[64] Francolini I., Norris P., Piozzi A., Donelli G., Stoodley P. (2004). Usnic acid, a natural antimicrobial agent able to inhibit bacterial biofilm formation on polymer surfaces. Antimicrob. Agents Chemother..

[65] Nithyanand P., Shafreen R.M.B., Muthamil S., Pandian S.K. (2015). Usnic acid inhibits biofilm formation and virulent morphological traits of *Candida albicans*. Microbiol. Res..

[66] Park J.-H., Lee J.-H., Cho M.H., Herzberg M., Lee J. (2012). Acceleration of protease effect on *Staphylococcus aureus* biofilm dispersal. FEMS Microbiol. Lett..

[67] Kharidia R., Liang J.F. (2011). The activity of a small lytic peptide PTP-7 on *Staphylococcus aureus* biofilms. J. Microbiol..

[68] Cho J.H., Sung B.H., Kim S.C. (2009). Buforins: Histone H2A-derived antimicrobial peptides from toad stomach. Biochim. Biophys. Acta (BBA)-Biomembr..

[69] Subbalakshmi C., Sitaram N. (1998). Mechanism of antimicrobial action of indolicidin. FEMS Microbiol. Lett..

[70] De la Fuente-Núñez C., Reffuveille F., Haney E.F., Straus S.K., Hancock R.E. (2014). Broad-spectrum anti-biofilm peptide that targets a cellular stress response. PLoS Pathog..

[71] Heddle J.G., Blance S.J., Zamble D.B., Hollfelder F., Miller D.A., Wentzell L.M., Walsh C.T., Maxwell A. (2001). The antibiotic microcin B17 is a DNA gyrase poison: Characterisation of the mode of inhibition. J. Mol. Biol..

[72] Kim J.-Y., Park S.-C., Yoon M.-Y., Hahm K.-S., Park Y. (2011). C-terminal amidation of PMAP-23: Translocation to the inner membrane of Gram-negative bacteria. Amino Acids.

[73] Rendueles O., Kaplan J.B., Ghigo J.M. (2013). Antibiofilm polysaccharides. Environ. Microbiol..

[74] Qin Z., Yang L., Qu D., Molin S., Tolker-Nielsen T. (2009). *Pseudomonas aeruginosa* extracellular products inhibit staphylococcal growth, and disrupt established biofilms produced by *Staphylococcus epidermidis*. Microbiology.

[75] Liang Z.-X. (2015). The expanding roles of c-di-GMP in the biosynthesis of exopolysaccharides and secondary metabolites. Nat. Prod. Rep..

[76] Deokar A.R., Shalom Y., Perelshtein I., Perkas N., Gedanken A., Banin E. (2016). A topical antibacterial ointment made of Zn-doped copper oxide nanocomposite. J. Nanopart. Res..

[77] de Lacerda Coriolano D., de Souza J.B., Bueno E.V., Medeiros S.M.D.F.R.D.S., Cavalcanti I.D.L., Cavalcanti I.M.F. (2021). Antibacterial and antibiofilm potential of silver nanoparticles against antibiotic-sensitive and multidrug-resistant *Pseudomonas aeruginosa* strains. Braz. J. Microbiol..

[78] Qais F.A., Ahmad I., Altaf M., Alotaibi S.H. (2021). Biofabrication of gold nanoparticles using *Capsicum annuum* extract and its antiquorum sensing and antibiofilm activity against bacterial pathogens. ACS Omega.

[79] Sun L.-M., Zhang C.-L., Li P. (2012). Characterization, antibiofilm, and mechanism of action of novel PEG-stabilized lipid nanoparticles loaded with terpinen-4-ol. J. Agric. Food Chem..

[80] Deryabin D., Galadzhieva A., Kosyan D., Duskaev G. (2019). Plant-derived inhibitors of AHL-mediated quorum sensing in bacteria: Modes of action. Int. J. Mol. Sci..

[81] Asfour H.Z. (2018). Anti-quorum sensing natural compounds. J. Microsc. Ultrastruct..

[82] Di Somma A., Moretta A., Canè C., Cirillo A., Duilio A. (2020). Antimicrobial and antibiofilm peptides. Biomolecules.

[83] Arifiyanto A., Surtiningsih T., Agustina D., Alami N.H. (2020). Antimicrobial activity of biosurfactants produced by actinomycetes isolated from rhizosphere of Sidoarjo mud region. Biocatal. Agric. Biotechnol..

[84] Zomorodian K., Veisi H., Mousavi S.M., Ataabadi M.S., Yazdanpanah S., Bagheri J., Mehr A.P., Hemmati S., Veisi H. (2018). Modified magnetic nanoparticles by PEG-400-immobilized Ag nanoparticles (Fe_3_O_4_@ PEG–Ag) as a core/shell nanocomposite and evaluation of its antimicrobial activity. Int. J. Nanomed..

[85] Payne D.E., Martin N.R., Parzych K.R., Rickard A.H., Underwood A., Boles B.R. (2013). Tannic acid inhibits *Staphylococcus aureus* surface colonization in an IsaA-dependent manner. Infect. Immun..

[86] Shen Y., Köller T., Kreikemeyer B., Nelson D.C. (2013). Rapid degradation of *Streptococcus pyogenes* biofilms by PlyC, a bacteriophage-encoded endolysin. J. Antimicrob. Chemother..

[87] Kragol G., Hoffmann R., Chattergoon M.A., Lovas S., Cudic M., Bulet P., Condie B.A., Rosengren K.J., Montaner L.J., Otvos L. (2002). Identification of crucial residues for the antibacterial activity of the proline-rich peptide, pyrrhocoricin. Eur. J. Biochem..

[88] Pierrat O.A., Maxwell A. (2005). Evidence for the role of DNA strand passage in the mechanism of action of microcin B17 on DNA gyrase. Biochemistry.

[89] Debebe T., Krüger M., Huse K., Kacza J., Mühlberg K., König B., Birkenmeier G. (2016). Ethyl pyruvate: An anti-microbial agent that selectively targets Pathobionts and biofilms. PLoS ONE.

[90] Yu C., Li X., Zhang N., Wen D., Liu C., Li Q. (2016). Inhibition of biofilm formation by D-tyrosine: Effect of bacterial type and D-tyrosine concentration. Water Res..

[91] Boles B.R., Horswill A.R. (2011). Staphylococcal biofilm disassembly. Trends Microbiol..

[92] Jiang P., Li J., Han F., Duan G., Lu X., Gu Y., Yu W. (2011). Antibiofilm activity of an exopolysaccharide from marine bacterium *Vibrio* sp. QY101. PLoS ONE.

[93] Yu S., Su T., Wu H., Liu S., Wang D., Zhao T., Jin Z., Du W., Zhu M.-J., Chua S.L. (2015). PslG, a self-produced glycosyl hydrolase, triggers biofilm disassembly by disrupting exopolysaccharide matrix. Cell Res..

[94] Wu S., Liu G., Jin W., Xiu P., Sun C. (2016). Antibiofilm and anti-infection of a marine bacterial exopolysaccharide against *Pseudomonas aeruginosa*. Front. Microbiol..

[95] de la Fuente-Núñez C., Korolik V., Bains M., Nguyen U., Breidenstein E.B., Horsman S., Lewenza S., Burrows L., Hancock R.E. (2012). Inhibition of bacterial biofilm formation and swarming motility by a small synthetic cationic peptide. Antimicrob. Agents Chemother..

[96] Lahiri D., Nag M., Dey A., Sarkar T., Ray R.R., Rebezov M., Shariati M.A., Thiruvengadam M., Simal-Gandara J. (2022). Immobilized enzymes as potent antibiofilm agent. Biotechnol. Prog..

[97] Mogi T., Kita K. (2009). Gramicidin S and polymyxins: The revival of cationic cyclic peptide antibiotics. Cell. Mol. Life Sci..

[98] Schaefer A.L., Hanzelka B.L., Eberhard A., Greenberg E. (1996). Quorum sensing in *Vibrio* fischeri: Probing autoinducer-LuxR interactions with autoinducer analogs. J. Bacteriol..

[99] Reverchon S., Chantegrel B., Deshayes C., Doutheau A., Cotte-Pattat N. (2002). New synthetic analogues of N-acyl homoserine lactones as agonists or antagonists of transcriptional regulators involved in bacterial quorum sensing. Bioorg. Med. Chem. Lett..

[100] Geske G.D., Mattmann M.E., Blackwell H.E. (2008). Evaluation of a focused library of N-aryl L-homoserine lactones reveals a new set of potent quorum sensing modulators. Bioorg. Med. Chem. Lett..

[101] Geske G.D., O’Neill J.C., Miller D.M., Mattmann M.E., Blackwell H.E. (2007). Modulation of bacterial quorum sensing with synthetic ligands: Systematic evaluation of N-acylated homoserine lactones in multiple species and new insights into their mechanisms of action. J. Am. Chem. Soc..

[102] Passador L., Tucker K.D., Guertin K.R., Journet M.P., Kende A.S., Iglewski B.H. (1996). Functional analysis of the *Pseudomonas aeruginosa* autoinducer PAI. J. Bacteriol..

[103] Ni N., Li M., Wang J., Wang B. (2009). Inhibitors and antagonists of bacterial quorum sensing. Med. Res. Rev..

[104] Castang S., Chantegrel B., Deshayes C., Dolmazon R., Gouet P., Haser R., Reverchon S., Nasser W., Hugouvieux-Cotte-Pattat N., Doutheau A. (2004). N-Sulfonyl homoserine lactones as antagonists of bacterial quorum sensing. Bioorg. Med. Chem. Lett..

[105] Boukraa M., Sabbah M., Soulère L., El Efrit M.L., Queneau Y., Doutheau A. (2011). AHL-dependent quorum sensing inhibition: Synthesis and biological evaluation of α-(N-alkyl-carboxamide)-γ-butyrolactones and α-(N-alkyl-sulfonamide)-γ-butyrolactones. Bioorg. Med. Chem. Lett..

[106] Brackman G., Risseeuw M., Celen S., Cos P., Maes L., Nelis H.J., Van Calenbergh S., Coenye T. (2012). Synthesis and evaluation of the quorum sensing inhibitory effect of substituted triazolyldihydrofuranones. Bioorg. Med. Chem..

[107] Morohoshi T., Shiono T., Takidouchi K., Kato M., Kato N., Kato J., Ikeda T. (2007). Inhibition of quorum sensing in *Serratia marcescens* AS-1 by synthetic analogs of N-acylhomoserine lactone. Appl. Environ. Microbiol..

[108] Ishida T., Ikeda T., Takiguchi N., Kuroda A., Ohtake H., Kato J. (2007). Inhibition of quorum sensing in *Pseudomonas aeruginosa* by N-acyl cyclopentylamides. Appl. Environ. Microbiol..

[109] Geske G.D., Wezeman R.J., Siegel A.P., Blackwell H.E. (2005). Small molecule inhibitors of bacterial quorum sensing and biofilm formation. J. Am. Chem. Soc..

[110] Girennavar B., Cepeda M.L., Soni K.A., Vikram A., Jesudhasan P., Jayaprakasha G., Pillai S.D., Patil B.S. (2008). Grapefruit juice and its furocoumarins inhibits autoinducer signaling and biofilm formation in bacteria. Int. J. Food Microbiol..

[111] Adonizio A., Kong K.-F., Mathee K. (2008). Inhibition of quorum sensing-controlled virulence factor production in *Pseudomonas aeruginosa* by South Florida plant extracts. Antimicrob. Agents Chemother..

[112] Rutherford S.T., Bassler B.L. (2012). Bacterial quorum sensing: Its role in virulence and possibilities for its control. Cold Spring Harb. Perspect. Med..

[113] Schuster M., Joseph Sexton D., Diggle S.P., Peter Greenberg E. (2013). Acyl-homoserine lactone quorum sensing: From evolution to application. Annu. Rev. Microbiol..

[114] Gambello M.J., Iglewski B.H. (1991). Cloning and characterization of the *Pseudomonas aeruginosa* lasR gene, a transcriptional activator of elastase expression. J. Bacteriol..

[115] Passador L., Cook J.M., Gambello M.J., Rust L., Iglewski B.H. (1993). Expression of *Pseudomonas aeruginosa* virulence genes requires cell-to-cell communication. Science.

[116] Givskov M., de Nys R., Manefield M., Gram L., Maximilien R., Eberl L., Molin S., Steinberg P.D., Kjelleberg S. (1996). Eukaryotic interference with homoserine lactone-mediated prokaryotic signalling. J. Bacteriol..

[117] Manefield M., de Nys R., Naresh K., Roger R., Givskov M., Peter S., Kjelleberg S. (1999). Evidence that halogenated furanones from *Delisea pulchra* inhibit acylated homoserine lactone (AHL)-mediated gene expression by displacing the AHL signal from its receptor protein. Microbiology.

[118] Hentzer M., Wu H., Andersen J.B., Riedel K., Rasmussen T.B., Bagge N., Kumar N., Schembri M.A., Song Z., Kristoffersen P. (2003). Attenuation of *Pseudomonas aeruginosa* virulence by quorum sensing inhibitors. EMBO J..

[119] Manefield M., Harris L., Rice S.A., de Nys R., Kjelleberg S. (2000). Inhibition of luminescence and virulence in the black tiger prawn (*Penaeus monodon*) pathogen *Vibrio harveyi* by intercellular signal antagonists. Appl. Environ. Microbiol..

[120] Huber B., Eberl L., Feucht W., Polster J. (2003). Influence of polyphenols on bacterial biofilm formation and quorum-sensing. Z. Nat. C.

[121] Lee J.-H., Park J.-H., Cho H.S., Joo S.W., Cho M.H., Lee J. (2013). Anti-biofilm activities of quercetin and tannic acid against *Staphylococcus aureus*. Biofouling.

[122] Manner S., Skogman M., Goeres D., Vuorela P., Fallarero A. (2013). Systematic exploration of natural and synthetic flavonoids for the inhibition of *Staphylococcus aureus* biofilms. Int. J. Mol. Sci..

[123] Kali A., Devaraj Bhuvaneshwar P., Charles M., Seetha K.S. (2016). Antibacterial synergy of curcumin with antibiotics against biofilm producing clinical bacterial isolates. J. Basic Clin. Pharm..

[124] Rasmussen T.B., Bjarnsholt T., Skindersoe M.E., Hentzer M., Kristoffersen P., Köte M., Nielsen J., Eberl L., Givskov M. (2005). Screening for quorum-sensing inhibitors (QSI) by use of a novel genetic system, the QSI selector. J. Bacteriol..

[125] Rasmussen T.B., Skindersoe M.E., Bjarnsholt T., Phipps R.K., Christensen K.B., Jensen P.O., Andersen J.B., Koch B., Larsen T.O., Hentzer M. (2005). Identity and effects of quorum-sensing inhibitors produced by *Penicillium* species. Microbiology.

[126] Nakamoto M., Kunimura K., Suzuki J.I., Kodera Y. (2020). Antimicrobial properties of hydrophobic compounds in garlic: *Allicin, vinyldithiin*, ajoene and diallyl polysulfides. Exp. Ther. Med..

[127] Bjarnsholt T., Jensen P.Ø., Rasmussen T.B., Christophersen L., Calum H., Hentzer M., Hougen H.-P., Rygaard J., Moser C., Eberl L. (2005). Garlic blocks quorum sensing and promotes rapid clearing of pulmonary *Pseudomonas aeruginosa* infections. Microbiology.

[128] Jakobsen T.H., van Gennip M., Phipps R.K., Shanmugham M.S., Christensen L.D., Alhede M., Skindersoe M.E., Rasmussen T.B., Friedrich K., Uthe F. (2012). Ajoene, a sulfur rich molecule from garlic, inhibits genes controlled by quorum sensing. Antimicrob. Agents Chemother..

[129] Brackman G., Hillaert U., Van Calenbergh S., Nelis H.J., Coenye T. (2009). Use of quorum sensing inhibitors to interfere with biofilm formation and development in *Burkholderia multivorans* and *Burkholderia cenocepacia*. Res. Microbiol..

[130] Brackman G., Cos P., Maes L., Nelis H.J., Coenye T. (2011). Quorum sensing inhibitors increase the susceptibility of bacterial biofilms to antibiotics in vitro and in vivo. Antimicrob. Agents Chemother..

[131] Li J., Koh J.-J., Liu S., Lakshminarayanan R., Verma C.S., Beuerman R.W. (2017). Membrane active antimicrobial peptides: Translating mechanistic insights to design. Front. Neurosci..

[132] Bierbaum G., Sahl H.-G. (2009). Lantibiotics: Mode of action, biosynthesis and bioengineering. Curr. Pharm. Biotechnol..

[133] Hasper H.E., Kramer N.E., Smith J.L., Hillman J., Zachariah C., Kuipers O.P., De Kruijff B., Breukink E. (2006). An alternative bactericidal mechanism of action for lantibiotic peptides that target lipid II. Science.

[134] Hsu S.-T.D., Breukink E., Tischenko E., Lutters M.A., de Kruijff B., Kaptein R., Bonvin A.M., van Nuland N.A. (2004). The nisin–lipid II complex reveals a pyrophosphate cage that provides a blueprint for novel antibiotics. Nat. Struct. Mol. Biol..

[135] Merghni A., Dallel I., Noumi E., Kadmi Y., Hentati H., Tobji S., Amor A.B., Mastouri M. (2017). Antioxidant and antiproliferative potential of biosurfactants isolated from *Lactobacillus casei* and their anti-biofilm effect in oral *Staphylococcus aureus* strains. Microb. Pathog..

[136] Dalili D., Amini M., Faramarzi M.A., Fazeli M.R., Khoshayand M.R., Samadi N. (2015). Isolation and structural characterization of Coryxin, a novel cyclic lipopeptide from *Corynebacterium xerosis* NS5 having emulsifying and anti-biofilm activity. Colloids Surf. B Biointerfaces.

[137] Balan S.S., Kumar C.G., Jayalakshmi S. (2016). Pontifactin, a new lipopeptide biosurfactant produced by a marine *Pontibacter korlensis* strain SBK-47: Purification, characterization and its biological evaluation. Process Biochem..

[138] Elshikh M., Funston S., Chebbi A., Ahmed S., Marchant R., Banat I.M. (2017). Rhamnolipids from non-pathogenic *Burkholderia thailandensis* E264: Physicochemical characterization, antimicrobial and antibiofilm efficacy against oral hygiene related pathogens. New Biotechnol..

[139] Abdollahi S., Tofighi Z., Babaee T., Shamsi M., Rahimzadeh G., Rezvanifar H., Saeidi E., Amiri M.M., Ashtiani Y.S., Samadi N. (2020). Evaluation of Anti-oxidant and Anti-biofilm Activities of Biogenic Surfactants Derived from *Bacillus amyloliquefaciens* and *Pseudomonas aeruginosa*. Iran. J. Pharm. Res. IJPR.

[140] Moryl M., Spętana M., Dziubek K., Paraszkiewicz K., Różalska S., Płaza G.A., Różalski A. (2015). Antimicrobial, antiadhesive and antibiofilm potential of lipopeptides synthesised by *Bacillus subtilis*, on uropathogenic bacteria. Acta Biochim. Pol..

[141] Karlapudi A.P., Venkateswarulu T., Srirama K., Kota R.K., Mikkili I., Kodali V.P. (2020). Evaluation of anti-cancer, anti-microbial and anti-biofilm potential of biosurfactant extracted from an *Acinetobacter* M6 strain. J. King Saud Univ.-Sci..

[142] Morais I., Cordeiro A., Teixeira G., Domingues V., Nardi R., Monteiro A., Alves R., Siqueira E., Santos V. (2017). Biological and physicochemical properties of biosurfactants produced by *Lactobacillus jensenii* P 6A and *Lactobacillus gasseri* P 65. Microb. Cell Factories.

[143] Shah I.M., Laaberki M.-H., Popham D.L., Dworkin J. (2008). A eukaryotic-like Ser/Thr kinase signals bacteria to exit dormancy in response to peptidoglycan fragments. Cell.

[144] Fischetti V.A. (2010). Bacteriophage endolysins: A novel anti-infective to control Gram-positive pathogens. Int. J. Med. Microbiol..

[145] Hoopes J.T., Stark C.J., Kim H.A., Sussman D.J., Donovan D.M., Nelson D.C. (2009). Use of a bacteriophage lysin, PlyC, as an enzyme disinfectant against *Streptococcus equi*. Appl. Environ. Microbiol..

[146] Köller T., Nelson D., Nakata M., Kreutzer M., Fischetti V.A., Glocker M.O., Podbielski A., Kreikemeyer B. (2008). PlyC, a novel bacteriophage lysin for compartment-dependent proteomics of group A streptococci. Proteomics.

[147] McGowan S., Buckle A.M., Mitchell M.S., Hoopes J.T., Gallagher D.T., Heselpoth R.D., Shen Y., Reboul C.F., Law R.H., Fischetti V.A. (2012). X-ray crystal structure of the streptococcal specific phage lysin PlyC. Proc. Natl. Acad. Sci. USA.

[148] Nelson D., Schuch R., Chahales P., Zhu S., Fischetti V.A. (2006). PlyC: A multimeric bacteriophage lysin. Proc. Natl. Acad. Sci. USA.

[149] Yoda Y., Hu Z.-Q., Zhao W.-H., Shimamura T. (2004). Different susceptibilities of *Staphylococcus* and Gram-negative rods to epigallocatechin gallate. J. Infect. Chemother..

[150] Zhao W.-H., Hu Z.-Q., Hara Y., Shimamura T. (2002). Inhibition of penicillinase by epigallocatechin gallate resulting in restoration of antibacterial activity of penicillin against penicillinase-producing *Staphylococcus aureus*. Antimicrob. Agents Chemother..

[151] Carpentier B., Cerf O. (1993). Biofilms and their consequences, with particular reference to hygiene in the food industry. J. Appl. Bacteriol..

[152] Stapleton M.R., Horsburgh M.J., Hayhurst E.J., Wright L., Jonsson M., Tarkowski A., Kokai-Kun J.F., Mond J.J., Foster S.J. (2007). Characterization of IsaA and SceD, two putative lytic transglycosylases of *Staphylococcus aureus*. J. Bacteriol..

[153] Höltje J., Mirelman D., Sharon N., Schwarz U. (1975). Novel type of murein transglycosylase in *Escherichia coli*. J. Bacteriol..

[154] Kragol G., Lovas S., Varadi G., Condie B.A., Hoffmann R., Otvos L. (2001). The antibacterial peptide pyrrhocoricin inhibits the ATPase actions of DnaK and prevents chaperone-assisted protein folding. Biochemistry.

[155] Gagnon M.G., Roy R.N., Lomakin I.B., Florin T., Mankin A.S., Steitz T.A. (2016). Structures of proline-rich peptides bound to the ribosome reveal a common mechanism of protein synthesis inhibition. Nucleic Acids Res..

[156] Kaplan J.Á. (2010). Biofilm dispersal: Mechanisms, clinical implications, and potential therapeutic uses. J. Dent. Res..

[157] Saggu S.K., Jha G., Mishra P.C. (2019). Enzymatic degradation of biofilm by metalloprotease from *Microbacterium* sp. SKS10. Front. Bioeng. Biotechnol..

[158] Abee T., Kovács Á.T., Kuipers O.P., Van der Veen S. (2011). Biofilm formation and dispersal in Gram-positive bacteria. Curr. Opin. Biotechnol..

[159] Thoendel M., Kavanaugh J.S., Flack C.E., Horswill A.R. (2010). Peptide signaling in the staphylococci. Chem. Rev..

[160] Beenken K.E., Mrak L.N., Griffin L.M., Zielinska A.K., Shaw L.N., Rice K.C., Horswill A.R., Bayles K.W., Smeltzer M.S. (2010). Epistatic relationships between sarA and agr in *Staphylococcus aureus* biofilm formation. PLoS ONE.

[161] Tsang L.H., Cassat J.E., Shaw L.N., Beenken K.E., Smeltzer M.S. (2008). Factors contributing to the biofilm-deficient phenotype of *Staphylococcus aureus* sarA mutants. PLoS ONE.

[162] Lauderdale K.J., Boles B.R., Cheung A.L., Horswill A.R. (2009). Interconnections between Sigma B, agr, and proteolytic activity in *Staphylococcus aureus* biofilm maturation. Infect. Immun..

[163] Mann E.E., Rice K.C., Boles B.R., Endres J.L., Ranjit D., Chandramohan L., Tsang L.H., Smeltzer M.S., Horswill A.R., Bayles K.W. (2009). Modulation of eDNA release and degradation affects *Staphylococcus aureus* biofilm maturation. PLoS ONE.

[164] Izano E.A., Amarante M.A., Kher W.B., Kaplan J.B. (2008). Differential roles of poly-N-acetylglucosamine surface polysaccharide and extracellular DNA in *Staphylococcus aureus* and *Staphylococcus epidermidis* biofilms. Appl. Environ. Microbiol..

[165] Romero D., Kolter R. (2011). Will biofilm disassembly agents make it to market?. Trends Microbiol..

[166] Zhou Y., Blanco L.P., Smith D.R., Chapman M.R. (2012). Bacterial amyloids. Amyloid Proteins.

[167] Barnhart M.M., Chapman M.R. (2006). Curli biogenesis and function. Annu. Rev. Microbiol..

[168] Cegelski L., Pinkner J.S., Hammer N.D., Cusumano C.K., Hung C.S., Chorell E., Åberg V., Walker J.N., Seed P.C., Almqvist F. (2009). Small-molecule inhibitors target *Escherichia coli* amyloid biogenesis and biofilm formation. Nat. Chem. Biol..

[169] Connolly K.L., Roberts A.L., Holder R.C., Reid S.D. (2011). Dispersal of Group A streptococcal biofilms by the cysteine protease SpeB leads to increased disease severity in a murine model. PLoS ONE.

[170] Bhoopalan S.V., Piekarowicz A., Lenz J.D., Dillard J.P., Stein D.C. (2016). nagZ triggers gonococcal biofilm disassembly. Sci. Rep..

[171] Das T., Manefield M. (2012). Pyocyanin promotes extracellular DNA release in *Pseudomonas aeruginosa*. PLoS ONE.

[172] Pihl M., Davies J.R., Chávez de Paz L.E., Svensäter G. (2010). Differential effects of *Pseudomonas aeruginosa* on biofilm formation by different strains of *Staphylococcus epidermidis*. FEMS Immunol. Med. Microbiol..

[173] Valle J., Da Re S., Henry N., Fontaine T., Balestrino D., Latour-Lambert P., Ghigo J.-M. (2006). Broad-spectrum biofilm inhibition by a secreted bacterial polysaccharide. Proc. Natl. Acad. Sci. USA.

[174] Bendaoud M., Vinogradov E., Balashova N.V., Kadouri D.E., Kachlany S.C., Kaplan J.B. (2011). Broad-spectrum biofilm inhibition by *Kingella kingae* exopolysaccharide. J. Bacteriol..

[175] Potrykus K., Cashel M. (2008). (p) ppGpp: Still magical?. Annu. Rev. Microbiol..

[176] Reffuveille F., de la Fuente-Núñez C., Mansour S., Hancock R.E. (2014). A broad-spectrum antibiofilm peptide enhances antibiotic action against bacterial biofilms. Antimicrob. Agents Chemother..

[177] Lemos J.A., Brown T.A., Burne R.A. (2004). Effects of RelA on key virulence properties of planktonic and biofilm populations of *Streptococcus mutans*. Infect. Immun..

[178] Römling U., Galperin M.Y., Gomelsky M. (2013). Cyclic di-GMP: The first 25 years of a universal bacterial second messenger. Microbiol. Mol. Biol. Rev..

[179] Chua S.L., Liu Y., Yam J.K.H., Chen Y., Vejborg R.M., Tan B.G.C., Kjelleberg S., Tolker-Nielsen T., Givskov M., Yang L. (2014). Dispersed cells represent a distinct stage in the transition from bacterial biofilm to planktonic lifestyles. Nat. Commun..

[180] Sambanthamoorthy K., Luo C., Pattabiraman N., Feng X., Koestler B., Waters C.M., Palys T.J. (2014). Identification of small molecules inhibiting diguanylate cyclases to control bacterial biofilm development. Biofouling.

[181] Kaplan J.B., Mlynek K.D., Hettiarachchi H., Alamneh Y.A., Biggemann L., Zurawski D.V., Black C.C., Bane C.E., Kim R.K., Granick M.S. (2018). Extracellular polymeric substance (EPS)-degrading enzymes reduce staphylococcal surface attachment and biocide resistance on pig skin in vivo. PLoS ONE.

[182] Kaplan J.B. (2009). Therapeutic potential of biofilm-dispersing enzymes. Int. J. Artif. Organs.

[183] Darouiche R.O., Mansouri M.D., Gawande P.V., Madhyastha S. (2009). Antimicrobial and antibiofilm efficacy of triclosan and DispersinB® combination. J. Antimicrob. Chemother..

[184] Dieltjens L., Appermans K., Lissens M., Lories B., Kim W., Van der Eycken E.V., Foster K.R., Steenackers H.P. (2020). Inhibiting bacterial cooperation is an evolutionarily robust anti-biofilm strategy. Nat. Commun..

[185] Pletzer D., Coleman S.R., Hancock R.E. (2016). Anti-biofilm peptides as a new weapon in antimicrobial warfare. Curr. Opin. Microbiol..

[186] Bahar A.A., Ren D. (2013). Antimicrobial peptides. Pharmaceuticals.

[187] Trimble M.J., Mlynárčik P., Kolář M., Hancock R.E. (2016). Polymyxin: Alternative mechanisms of action and resistance. Cold Spring Harb. Perspect. Med..

[188] Falanga A., Nigro E., De Biasi M.G., Daniele A., Morelli G., Galdiero S., Scudiero O. (2017). Cyclic peptides as novel therapeutic microbicides: Engineering of human defensin mimetics. Molecules.

[189] Cowan M.M. (1999). Plant products as antimicrobial agents. Clin. Microbiol. Rev..

[190] Molan P.C. (2001). Honey as a topical antibacterial agent for treatment of infected wounds. World Wide Wounds.

[191] Maddocks S.E., Lopez M.S., Rowlands R.S., Cooper R.A. (2012). Manuka honey inhibits the development of *Streptococcus pyogenes* biofilms and causes reduced expression of two fibronectin binding proteins. Microbiology.

[192] Ng W.-J., Lim K.-Y., Chong J.-Y., Low K.-L. (2014). In vitro screening of honey against *Enterococcus* spp. biofilm. J. Med. Bioeng..

[193] Lee J.-H., Park J.-H., Kim J.-A., Neupane G.P., Cho M.H., Lee C.-S., Lee J. (2011). Low concentrations of honey reduce biofilm formation, quorum sensing, and virulence in *Escherichia coli* O157: H7. Biofouling.

[194] Santangelo E. (2013). Honey. http://flipper.diff.org/app/items/info/4617.

[195] Nassar H.M., Li M., Gregory R.L. (2012). Effect of honey on *Streptococcus mutans* growth and biofilm formation. Appl. Environ. Microbiol..

[196] Ahmad I., Husain F.M., Maheshwari M., Zahin M. (2014). Medicinal plants and phytocompounds: A potential source of novel antibiofilm agents. Antibiofilm Agents.

[197] Berde C.V., Salvi S.P., Rawool P.P., Prathyusha A., Berde V.B. (2019). Role of medicinal plants and endophytic bacteria of medicinal plants in inhibition of biofilm formation: Interference in quorum sensing. Implication of Quorum Sensing and Biofilm Formation in Medicine, Agriculture and Food Industry.

[198] Ravichandiran V., Shanmugam K., Anupama K., Thomas S., Princy A. (2012). Structure-based virtual screening for plant-derived SdiA-selective ligands as potential antivirulent agents against uropathogenic *Escherichia coli*. Eur. J. Med. Chem..

[199] Zou J., Liu Y., Guo R., Tang Y., Shi Z., Zhang M., Wu W., Chen Y., Hou K. (2021). An In Vitro Coumarin-Antibiotic Combination Treatment of *Pseudomonas aeruginosa* Biofilms. Nat. Prod. Commun..

[200] Dey P., Parai D., Banerjee M., Hossain S.T., Mukherjee S.K. (2020). Naringin sensitizes the antibiofilm effect of ciprofloxacin and tetracycline against *Pseudomonas aeruginosa* biofilm. Int. J. Med. Microbiol..

[201] Liu Y., Xu Y., Song Q., Wang F., Sun L., Liu L., Yang X., Yi J., Bao Y., Ma H. (2017). Anti-biofilm activities from *Bergenia crassifolia* leaves against *Streptococcus mutans*. Front. Microbiol..

[202] Ouyang P., He X., Yuan Z.-W., Yin Z.-Q., Fu H., Lin J., He C., Liang X., Lv C., Shu G. (2018). Erianin against *Staphylococcus aureus* infection via inhibiting sortase A. Toxins.

[203] Zhou J.-W., Hou B., Liu G.-Y., Jiang H., Sun B., Wang Z.-N., Shi R.-F., Xu Y., Wang R., Jia A.-Q. (2018). Attenuation of *Pseudomonas aeruginosa* biofilm by hordenine: A combinatorial study with aminoglycoside antibiotics. Appl. Microbiol. Biotechnol..

[204] Nadaf N.H., Parulekar R.S., Patil R.S., Gade T.K., Momin A.A., Waghmare S.R., Dhanavade M.J., Arvindekar A.U., Sonawane K.D. (2018). Biofilm inhibition mechanism from extract of *Hymenocallis littoralis* leaves. J. Ethnopharmacol..

[205] Kalia M., Yadav V.K., Singh P.K., Sharma D., Narvi S.S., Agarwal V. (2018). Exploring the impact of parthenolide as anti-quorum sensing and anti-biofilm agent against *Pseudomonas aeruginosa*. Life Sci..

[206] Fu B., Wu Q., Dang M., Bai D., Guo Q., Shen L., Duan K. (2017). Inhibition of *Pseudomonas aeruginosa* biofilm formation by traditional Chinese medicinal herb *Herba patriniae*. BioMed Res. Int..

[207] Lopes L.A.A., dos Santos Rodrigues J.B., Magnani M., de Souza E.L., de Siqueira-Júnior J.P. (2017). Inhibitory effects of flavonoids on biofilm formation by *Staphylococcus aureus* that overexpresses efflux protein genes. Microb. Pathog..

[208] Wang J., Song M., Pan J., Shen X., Liu W., Zhang X., Li H., Deng X. (2018). Quercetin impairs *Streptococcus pneumoniae* biofilm formation by inhibiting sortase A activity. J. Cell. Mol. Med..

[209] da Silva Trentin D., Giordani R.B., Zimmer K.R., Da Silva A.G., Da Silva M.V., dos Santos Correia M.T., Baumvol I.J.R., Macedo A.J. (2011). Potential of medicinal plants from the Brazilian semi-arid region (Caatinga) against *Staphylococcus epidermidis* planktonic and biofilm lifestyles. J. Ethnopharmacol..

[210] Faraz N.F.N., Zia-ul-Islam R.R. (2012). Antibiofilm forming activity of naturally occurring compound. Biomedica.

[211] Singh B.N., Singh H.B., Singh A., Singh B.R., Mishra A., Nautiyal C.S. (2012). *Lagerstroemia speciosa* fruit extract modulates quorum sensing-controlled virulence factor production and biofilm formation in *Pseudomonas aeruginosa*. Microbiology.

[212] Burt S. (2004). Essential oils: Their antibacterial properties and potential applications in foods—A review. J. Appl. Microbiol..

[213] Hammer K.A., Carson C.F., Riley T.V. (1999). Antimicrobial activity of essential oils and other plant extracts. J. Appl. Microbiol..

[214] Nuryastuti T., van der Mei H.C., Busscher H.J., Iravati S., Aman A.T., Krom B.P. (2009). Effect of cinnamon oil on icaA expression and biofilm formation by *Staphylococcus epidermidis*. Appl. Environ. Microbiol..

[215] de Oliveira M.M.M., Brugnera D.F., do Nascimento J.A., Batista N.N., Piccoli R.H. (2012). Cinnamon essential oil and cinnamaldehyde in the control of bacterial biofilms formed on stainless steel surfaces. Eur. Food Res. Technol..

[216] Gholamnezhad Z., Shakeri F., Saadat S., Ghorani V., Boskabady M.H. (2019). Clinical and experimental effects of *Nigella sativa* and its constituents on respiratory and allergic disorders. Avicenna J. Phytomed..

[217] Derakhshan S., Sattari M., Bigdeli M. (2010). Effect of cumin (Cuminum cyminum) seed essential oil on biofilm formation and plasmid Integrity of *Klebsiella pneumoniae*. Pharmacogn. Mag..

[218] Nostro A., Roccaro A.S., Bisignano G., Marino A., Cannatelli M.A., Pizzimenti F.C., Cioni P.L., Procopio F., Blanco A.R. (2007). Effects of oregano, carvacrol and thymol on *Staphylococcus aureus* and *Staphylococcus epidermidis* biofilms. J. Med. Microbiol..

[219] Oral N.B., Vatansever L., Aydin B.D., Sezer C., Guven A., Gumez M., Kurkcuoglu M. (2010). Effect of oregano essential oil on biofilms formed by Staphylococci and *Escherichia coli*. Kafkas Univ. Vet. Fak. Derg..

[220] Coelho F.A.B.L., Lopes S.P., Pereira M.O. (2012). Effective Association of Tea Tree Essential Oil with Conventional Antibiotics to Control Pseudomonas aeruginosa Biofilms. https://hdl.handle.net/1822/28611.

[221] Szczepanski S., Lipski A. (2014). Essential oils show specific inhibiting effects on bacterial biofilm formation. Food Control.

[222] Brady A., Loughlin R., Gilpin D., Kearney P., Tunney M. (2006). In vitro activity of tea-tree oil against clinical skin isolates of meticillin-resistant and-sensitive *Staphylococcus aureus* and coagulase-negative staphylococci growing planktonically and as biofilms. J. Med. Microbiol..

[223] Filogônio C.D.F.B., Soares R.V., Horta M.C.R., Penido C.V.D.S.R., Cruz R.D.A. (2011). Effect of vegetable oil (Brazil nut oil) and mineral oil (liquid petrolatum) on dental biofilm control. Braz. Oral Res..

[224] Jackson K.D., Starkey M., Kremer S., Parsek M.R., Wozniak D.J. (2004). Identification of psl, a locus encoding a potential exopolysaccharide that is essential for *Pseudomonas aeruginosa* PAO1 biofilm formation. J. Bacteriol..

[225] DiGiandomenico A., Warrener P., Hamilton M., Guillard S., Ravn P., Minter R., Camara M.M., Venkatraman V., MacGill R.S., Lin J. (2012). Identification of broadly protective human antibodies to *Pseudomonas aeruginosa* exopolysaccharide Psl by phenotypic screening. J. Exp. Med..

[226] Maira-Litrán T., Kropec A., Goldmann D.A., Pier G.B. (2005). Comparative opsonic and protective activities of *Staphylococcus aureus* conjugate vaccines containing native or deacetylated staphylococcal poly-N-acetyl-β-(1-6)-glucosamine. Infect. Immun..

[227] Algburi A., Comito N., Kashtanov D., Dicks L.M., Chikindas M.L. (2017). Control of biofilm formation: Antibiotics and beyond. Appl. Environ. Microbiol..

[228] Li C., Zhang X., Huang X., Wang X., Liao G., Chen Z. (2013). Preparation and characterization of flexible nanoliposomes loaded with daptomycin, a novel antibiotic, for topical skin therapy. Int. J. Nanomed..

[229] Suci P.A., Berglund D.L., Liepold L., Brumfield S., Pitts B., Davison W., Oltrogge L., Hoyt K.O., Codd S., Stewart P.S. (2007). High-density targeting of a viral multifunctional nanoplatform to a pathogenic, biofilm-forming bacterium. Chem. Biol..

[230] Taylor E., Webster T.J. (2011). Reducing infections through nanotechnology and nanoparticles. Int. J. Nanomed..

[231] Morones J.R., Elechiguerra J.L., Camacho A., Holt K., Kouri J.B., Ramírez J.T., Yacaman M.J. (2005). The bactericidal effect of silver nanoparticles. Nanotechnology.

[232] El Badawy A.M., Silva R.G., Morris B., Scheckel K.G., Suidan M.T., Tolaymat T.M. (2010). Surface charge-dependent toxicity of silver nanoparticles. Environ. Sci. Technol..

[233] Pal S., Tak Y.K., Song J.M. (2007). Does the antibacterial activity of silver nanoparticles depend on the shape of the nanoparticle? A study of the gram-negative bacterium *Escherichia coli*. Appl. Environ. Microbiol..

[234] Inbakandan D., Kumar C., Abraham L.S., Kirubagaran R., Venkatesan R., Khan S.A. (2013). Silver nanoparticles with anti microfouling effect: A study against marine biofilm forming bacteria. Colloids Surf. B Biointerfaces.

[235] Hajipour M.J., Fromm K.M., Ashkarran A.A., de Aberasturi D.J., de Larramendi I.R., Rojo T., Serpooshan V., Parak W.J., Mahmoudi M. (2012). Antibacterial properties of nanoparticles. Trends Biotechnol..

[236] Lemire J.A., Harrison J.J., Turner R.J. (2013). Antimicrobial activity of metals: Mechanisms, molecular targets and applications. Nat. Rev. Microbiol..

[237] Pelgrift R.Y., Friedman A.J. (2013). Nanotechnology as a therapeutic tool to combat microbial resistance. Adv. Drug Deliv. Rev..

[238] Subbiahdoss G., Sharifi S., Grijpma D.W., Laurent S., van der Mei H.C., Mahmoudi M., Busscher H.J. (2012). Magnetic targeting of surface-modified superparamagnetic iron oxide nanoparticles yields antibacterial efficacy against biofilms of gentamicin-resistant staphylococci. Acta Biomater..

[239] Sotiriou G.A., Pratsinis S.E. (2010). Antibacterial activity of nanosilver ions and particles. Environ. Sci. Technol..

[240] Sondi I., Salopek-Sondi B. (2004). Silver nanoparticles as antimicrobial agent: A case study on *E. coli* as a model for Gram-negative bacteria. J. Colloid Interface Sci..

[241] Egger S., Lehmann R.P., Height M.J., Loessner M.J., Schuppler M. (2009). Antimicrobial properties of a novel silver-silica nanocomposite material. Appl. Environ. Microbiol..

[242] Iavicoli I., Fontana L., Leso V., Bergamaschi A. (2013). The effects of nanomaterials as endocrine disruptors. Int. J. Mol. Sci..

[243] Hong R., Kang T.Y., Michels C.A., Gadura N. (2012). Membrane lipid peroxidation in copper alloy-mediated contact killing of *Escherichia coli*. Appl. Environ. Microbiol..

[244] Lee J., Ramasamy M. (2016). Recent nanotechnology approaches for prevention and treatment of biofilm-associated infections on medical devices recent nanotechnology approaches for prevention and treatment of biofilm-associated infections on medical devices. BioMed Res. Int..

[245] Baek Y.-W., An Y.-J. (2011). Microbial toxicity of metal oxide nanoparticles (CuO, NiO, ZnO, and Sb2O3) to *Escherichia coli*, *Bacillus subtilis*, and *Streptococcus aureus*. Sci. Total Environ..

[246] Afkhami F., Pourhashemi S.J., Sadegh M., Salehi Y., Fard M.J.K. (2015). Antibiofilm efficacy of silver nanoparticles as a vehicle for calcium hydroxide medicament against *Enterococcus faecalis*. J. Dent..

[247] Giri K., Yepes L.R., Duncan B., Parameswaran P.K., Yan B., Jiang Y., Bilska M., Moyano D.F., Thompson M.A., Rotello V.M. (2015). Targeting bacterial biofilms via surface engineering of gold nanoparticles. RSC Adv..

[248] Taheri S., Vasilev K., Majewski P. (2015). Silver nanoparticles: Synthesis, antimicrobial coatings, and applications for medical devices. Recent Pat. Mater. Sci..

[249] Tuby R., Gutfreund S., Perelshtein I., Mircus G., Ehrenberg M., Mimouni M., Gedanken A., Bahar I. (2016). Fabrication of a Stable and Efficient Antibacterial Nanocoating of Zn-CuO on Contact Lenses. Chem. NanoMat.

[250] Abdulkareem E.H., Memarzadeh K., Allaker R., Huang J., Pratten J., Spratt D. (2015). Anti-biofilm activity of zinc oxide and hydroxyapatite nanoparticles as dental implant coating materials. J. Dent..

[251] Ramachandran R., Sangeetha D. (2017). Antibiofilm efficacy of silver nanoparticles against biofilm forming multidrug resistant clinical isolates. Pharma Innov..

[252] Da Costa D., Exbrayat-Héritier C., Rambaud B., Megy S., Terreux R., Verrier B., Primard C. (2021). Surface charge modulation of rifampicin-loaded PLA nanoparticles to improve antibiotic delivery in *Staphylococcus aureus* biofilms. J. Nanobiotechnol..

[253] Anjugam M., Vaseeharan B., Iswarya A., Divya M., Prabhu N.M., Sankaranarayanan K. (2018). Biological synthesis of silver nanoparticles using β-1, 3 glucan binding protein and their antibacterial, antibiofilm and cytotoxic potential. Microb. Pathog..

[254] Ali S.G., Ansari M.A., Khan H.M., Jalal M., Mahdi A.A., Cameotra S.S. (2017). Crataeva nurvala nanoparticles inhibit virulence factors and biofilm formation in clinical isolates of *Pseudomonas aeruginosa*. J. Basic Microbiol..

[255] Amalaradjou M., Venkitanarayanan K. (2014). Antibiofilm effect of octenidine hydrochloride on *Staphylococcus aureus*, MRSA and VRSA. Pathogens.

[256] Rajput M., Bithel N., Vijayakumar S. (2021). Antimicrobial, antibiofilm, antioxidant, anticancer, and phytochemical composition of the seed extract of *Pongamia pinnata*. Arch. Microbiol..

[257] Caputo L., Capozzolo F., Amato G., De Feo V., Fratianni F., Vivenzio G., Nazzaro F. (2022). Chemical composition, antibiofilm, cytotoxic, and anti-acetylcholinesterase activities of *Myrtus communis* L. leaves essential oil. BMC Complementary Med. Ther..

[258] Valliammai A., Selvaraj A., Mathumitha P., Aravindraja C., Pandian S.K. (2021). Polymeric antibiofilm coating comprising synergistic combination of citral and thymol prevents methicillin-resistant *Staphylococcus aureus* biofilm formation on titanium. Mater. Sci. Eng. C.

[259] Ray Mohapatra A., Lakshmanan D., Mahesh R., Suchiang K., Jeevaratnam K. (2021). Characterization and Cytotoxic Evaluation of Bacteriocins Possessing Antibiofilm Activity Produced by *Lactobacillus plantarum* SJ33. Int. J. Pept. Res. Ther..

[260] Shakhatreh M.A., Al-Rawi O.F., Swedan S.F., Alzoubi K.H., Khabour O.F., Al-Fandi M. (2021). Biosynthesis of silver nanoparticles from *Citrobacter freundii* as antibiofilm agents with their cytotoxic effects on human cells. Curr. Pharm. Biotechnol..

[261] Patel M., Ashraf M.S., Siddiqui A.J., Ashraf S.A., Sachidanandan M., Snoussi M., Adnan M., Hadi S. (2020). Profiling and role of bioactive molecules from puntius sophore (Freshwater/brackish fish) skin mucus with its potent antibacterial, antiadhesion, and antibiofilm activities. Biomolecules.

[262] Singh G., Wani N.A., Rahim J.U., Shankar S., Rai R., Katoch M. (2022). Synergistic antimicrobial and antibiofilm activities of piperic acid and 4-ethylpiperic acid amides in combination with ciprofloxacin. J. Antibiot..

[263] Guchhait K.C., Manna T., Barai M., Karmakar M., Nandi S.K., Jana D., Dey A., Panda S., Raul P., Patra A. (2022). Antibiofilm and anticancer activities of unripe and ripe *Azadirachta indica* (neem) seed extracts. BMC Complementary Med. Ther..

[264] Cochis A., Barberi J., Ferraris S., Miola M., Rimondini L., Vernè E., Yamaguchi S., Spriano S. (2020). Competitive surface colonization of antibacterial and bioactive materials doped with strontium and/or silver ions. Nanomaterials.

[265] Percival S.L., Mayer D., Salisbury A.M. (2017). Efficacy of a surfactant-based wound dressing on biofilm control. Wound Repair Regen..

[266] Karaman D.Ş., Manner S., Fallarero A., Rosenholm J.M. (2017). Current approaches for exploration of nanoparticles as antibacterial agents. Antibacterial Agents.

[267] Malone M., Goeres D.M., Gosbell I., Vickery K., Jensen S., Stoodley P. (2017). Approaches to biofilm-associated infections: The need for standardized and relevant biofilm methods for clinical applications. Expert Rev. Anti-Infect. Ther..

[268] Hirsch T., Jacobsen F., Rittig A., Goertz O., Niederbichler A., Steinau H., Seipp H., Steinstraesser L. (2009). A comparative in vitro study of cell toxicity of clinically used antiseptics. Hautarzt Z. Dermatol. Venerol. Verwandte Geb..

[269] Cady N.C., McKean K.A., Behnke J., Kubec R., Mosier A.P., Kasper S.H., Burz D.S., Musah R.A. (2012). Inhibition of biofilm formation, quorum sensing and infection in *Pseudomonas aeruginosa* by natural products-inspired organosulfur compounds. PLoS ONE.

[270] Gopal R., Kim Y.G., Lee J.H., Lee S.K., Chae J.D., Son B.K., Seo C.H., Park Y. (2014). Synergistic effects and antibiofilm properties of chimeric peptides against multidrug-resistant *Acinetobacter baumannii* strains. Antimicrob. Agents Chemother..

[271] Ding Q., Tan K.S. (2016). The danger signal extracellular ATP is an inducer of *Fusobacterium nucleatum* biofilm dispersal. Front. Cell. Infect. Microbiol..

[272] Fleming D., Rumbaugh K.P. (2017). Approaches to dispersing medical biofilms. Microorganisms.

[273] Koo H., Allan R.N., Howlin R.P., Stoodley P., Hall-Stoodley L. (2017). Targeting microbial biofilms: Current and prospective therapeutic strategies. Nat. Rev. Microbiol..

